# Effectiveness of Alcohol Use Disorder Pharmacotherapies by Sex: Systematic Review and Meta‐Analysis

**DOI:** 10.1111/dar.70196

**Published:** 2026-06-23

**Authors:** Juliette Allen, Andrew Jones, Gillian W. Shorter, Catharine Montgomery, Zetta Kougiali, Alina Bagnall, Claire Adshead, Jessie Smith, Sam Burton, Amanda Atkinson, Lillian Guelen, Abigail Rose

**Affiliations:** ^1^ School of Psychology Liverpool John Moores University Liverpool UK; ^2^ School of Psychology Queen's University Belfast Belfast UK; ^3^ School of Nursing and Midwifery, Trinity College Dublin The University of Dublin Dublin Ireland; ^4^ TreAdd Research Group on Treatment and Addictions Tampere University Tampere Finland; ^5^ School of Medicine University of Nottingham Nottingham UK; ^6^ School of Psychology University of Derby Derby UK; ^7^ School of Psychology The University of Newcastle Newcastle Australia

**Keywords:** alcohol‐related disorders, meta‐analysis, pharmacotherapy, sex characteristics, treatment outcome

## Abstract

**Issues:**

Alcohol use disorder (AUD) shows sex‐related differences in prevalence, harm and treatment response. Despite growing interest in sex differences, evidence synthesis evaluating pharmacotherapy effectiveness by sex remains limited.

**Approach:**

Web of Science, PubMed, Scopus, PsycINFO and Cochrane were searched twice. Eligible records included RCTs or non‐randomised studies of adults with AUD receiving pharmacological interventions (licensed or off‐label), and reporting or providing outcomes (binary relapse or continuous alcohol consumption change) by sex. Multi‐level random‐effects models calculated risk ratios (RR) and standardised mean differences (SMD), with sex as a moderator.

**Key Findings:**

Twenty‐eight studies (25,041 participants, 25% female) were included. No outcomes were rated low risk of bias; non‐RCTs were moderate‐to‐high quality. Overall treatment effects vs. control were small for abstinence (RR = 0.96, 95% CI [0.52, 1.74]; 4 studies; *I* ^2^ = 95%) and modest for consumption reduction (SMD = 0.23, 95% CI [0.01, 0.45]; 13 studies; *I* ^2^ = 89%); sex did not meaningfully moderate these outcomes (ratio of RR = 1.04, 95% CI [0.87, 1.25]; ΔSMD = 0.05, 95% CI [−0.09, 0.18]). Power was low (median 13.7%), requiring ~6358 participants per group to detect the observed sex difference. Narrative synthesis suggested possible sex differences for naltrexone and baclofen, while highlighting the influence of drug (e.g., tolerability), participant (e.g., drinking motives) and design factors (e.g., recruitment setting) on treatment response.

**Implications:**

AUD pharmacotherapies provide modest benefits, with sex differences remaining unclear. Future trials should be adequately powered, report sex‐specific outcomes and consider adherence, tolerability and psychosocial moderators.

**Conclusion:**

Evidence for sex‐specific efficacy remains inconclusive. Patterns for naltrexone and baclofen warrant exploration in large, rigorously designed, sex‐stratified trials.

## Key Point Summary

- This systematic review and meta‐analysis exploring alcohol use disorder pharmacotherapy efficacy found modest overall effects, with no clear evidence for sex‐specific efficacy.
- The narrative synthesis indicated potential sex differences for naltrexone (greater efficacy in men) and baclofen (greater benefit in women).
- Study‐, drug‐ and participant‐related factors (e.g., tolerability, drinking motives, hormonal influences) may contribute to observed variability.
- Future trials should be adequately powered, report sex‐disaggregated outcomes and incorporate measures of biological and psychosocial moderators.

## 1 Introduction

Alcohol use disorder (AUD) is characterised by impaired control over alcohol consumption despite adverse consequences [1]. While overall consumption is declining across Europe [2], the impact of alcohol on health is rising [3, 4, 5]. In the United Kingdom (UK), alcohol‐specific deaths reached a record high in 2023, while alcohol‐related mortality increased by 39% in women and 38% in men between 2019 and 2023 [6, 7]. Similarly, from 1999 to 2020, the United States of America (USA) saw a 14.7% rise in alcohol‐related mortality for women and 12.5% for men [8].

Men typically consume more alcohol [2], leading to higher rates of alcohol‐related hospitalisations and fatalities [9, 10, 11]. However, women tend to be more vulnerable to the harmful effects of alcohol, even at lower consumption levels [12, 13]; they face increased risk of developing severe alcohol‐related liver disease, certain cancers, and cardiovascular disease despite lower alcohol intake [11, 14, 15]. National Survey on Drug Use and Health data (2009–2019) show that although overall AUD rates declined, this decline was significantly steeper among men than women [16]. Across health‐related pre‐clinical and clinical research, female animals and participants have historically been underrepresented due to concerns that hormonal fluctuations would introduce variability into the data [17, 18]. However, there is a rising demand for research to inform targeted treatments and support for women's alcohol use [19, 20].

Several mechanisms may underlie differences in pharmacotherapy effectiveness by sex. For example, female sex hormones (oestrogen and progesterone) can influence sensitivity to opioid antagonists with women experiencing differential subjective effects across their menstrual cycle [21, 22]. Sex differences in pharmacokinetics influence variations in peak drug concentration and bioavailability affecting the levels and duration of action of medications [23, 24]. A recent review found poorer tolerability and more severe side effects from acamprosate, naltrexone, baclofen and varenicline in women, potentially affecting adherence [25]. Gender‐related factors, such as differences in stress‐related drinking [26, 27] and early evidence suggesting that anxiolytic medications may be more effective in women [28], could impact treatment outcomes.

Despite these observations, literature on sex differences in AUD treatment response is limited and requires synthesis [29, 30] This systematic review and meta‐analysis aim to: (i) determine whether the effectiveness of AUD pharmacotherapies differs by sex; and (ii) identify whether mechanisms of therapeutic effect differ by sex.

## 2 Methods

### 2.1 Protocol and Registration

The review protocol was registered with the International Prospective Register of Systematic Reviews (PROSPERO) ID: CRD42023372885. The review adheres to the 2020 Preferred Reporting Items for Systematic Reviews and Meta‐analyses [31], as detailed in Table S3. Web of Science, PubMed, Scopus, PsycINFO and Cochrane were searched in May 2023 and updated in January 2025. The search strategy combined AUD population, pharmacological intervention, and treatment status terms; ‘AND’ was used to combine the three searches and identify relevant studies (Table S1). Sex and gender terms were not included in the search strategy to maximise study inclusion, as some trials report outcomes by sex/gender without indexing. Full texts were briefly assessed during early screening for sex‐disaggregated outcomes. This review uses the term ‘sex’ when discussing differences in pharmacotherapy response. Accordingly, the terms *female* and *male* are used as adjectives to describe trial participants throughout the results. However, gender, encompassing social, psychological and behavioural factors can influence treatment outcomes [32, 33]. Where appropriate, particularly when referring to population‐level trends or gendered experiences, the terms *women* and *men* are used as nouns.

### 2.2 Study Selection and Eligibility Criteria

Study selection and eligibility criteria targeted English‐language studies reporting AUD treatment outcomes by sex. Eligible studies included randomised controlled trials (RCT) and non‐randomised studies. Interventions were pharmacotherapies, licensed or unlicensed, for AUD treatment, excluding those used for managing alcohol withdrawal syndrome. A review of the literature and a recent meta‐analysis [34] identified 34 frequently prescribed pharmacotherapies for maintaining abstinence in AUD patients. Inclusion required baseline and/or post‐treatment alcohol use outcomes, such as changes in alcohol consumption quantity or frequency (Table 1).

**TABLE 1:** Eligibility criteria for study selection.

Population	Human participants
	Participants have alcohol use disorder^d^
	Participants are treatment‐seeking
Intervention	Any singular licensed pharmacotherapy for AUD^a^
	Any singular unlicensed pharmacotherapy for AUD^a^
	Any combination of pharmacotherapies for AUD^a^
Outcomes	Report alcohol consumption measure^b^
	Report baseline and/or post‐treatment alcohol‐related measure^c^ Report sex‐disaggregated data or sex‐by‐treatment analysis^e^
Design	Any type of randomised controlled trial
	Any type of observational study

Abbreviation: AUD, alcohol use disorder.

^a^ Acamprosate, amisulpride, aripiprazole, atenolol, baclofen, carbamazepine, citalopram/escitalopram, disulfiram, fluoxetine, flupenthixol, fluvoxamine, gabapentin, galantamine, gamma‐hydroxybutyric acid (GHB), levetiracetam, lisuride, lithium, memantine, modafinil nalmefene, naltrexone, nefazodone, ondansetron, oxcarbazepine, pregabalin, quetiapine, rimonabant, tianeptine, tiapride, topiramate, trazodone, varenicline.

^b^ Alcohol use: units p/week, units/drink p/day, *N* drinking days, frequency of drinking, *N* heavy drinking days (≥ 4 drinks per day for women; ≥ 5 for men), relapse (y/n).

^c^ Randomised controlled trial design requires post‐treatment outcome; observational design or studies with no comparator group require pre‐post outcome measure.

^d^ Due to changing Diagnostic and Statistical Manual of Mental Disorders terminology and not limits on publication dates, accepted terms include ‘alcohol‐dependent’ and ‘alcoholic’.

^e^ For meta‐analysis, studies were eligible if they provided sex‐disaggregated alcohol outcomes (e.g., means and SDs, or other measures that could be transformed); for review only, studies were eligible if they reported any form of sex‐related analysis (e.g., sex‐by‐treatment interactions, within‐sex comparisons across treatment arms).

**FIGURE 1:**
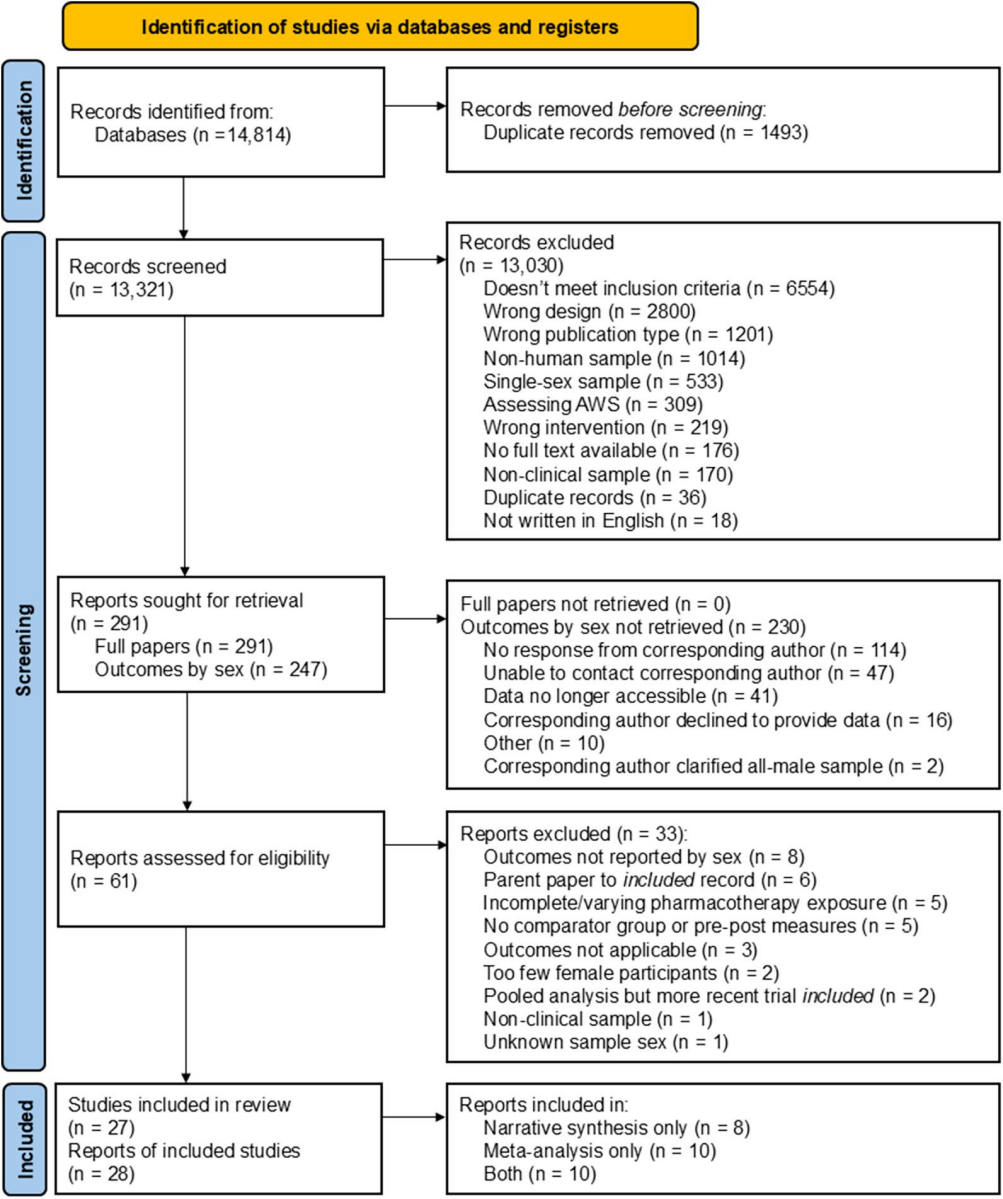
Preferred reporting items for systematic reviews and meta‐analyses (PRISMA) flow chart. The figure presents the PRISMA flow diagram detailing study identification, screening, eligibility assessment and final inclusion in the current systematic review and meta‐analysis. The initial database search yielded 14,814 records. After the removal of 1493 duplicates, 13,321 citations were eligible for title and abstract screening. This first phase of screening excluded of 13,030 records for various reasons: 6554 did not meet the main inclusion criteria 2800 used the wrong study design, 1201 were the wrong publication type, 1014 were not on human samples, 533 examined single‐sex samples, 309 assessed alcohol withdrawal syndrome (AWS), 219 concerned the wrong intervention, 176 lacked available full texts, 170 were on non‐clinical samples, 36 were newly identified duplicates, and 18 were not written in English. Following this, 291 reports were sought for retrieval for full‐text screening, comprising 44 readily available full texts and 247 reports for which the authors were contacted to provide sex‐specific outcome data. The second phase of screening excluded 33 reports for reasons including: Failure to report required outcomes by sex (*n* = 8), being a parent paper to an already included record (*n* = 6), having incomplete or varying pharmacotheraphy exposure (*n* = 5), reporting only non‐applicable outcomes (*n* = 3), having too few female participants (*n* = 2), being an older pooled analysis of an included record (*n* = 1), being on a non‐clinical sample (*n* = 1) and having unknown sample sex (*n* = 1). Ultimately, 28 records, representing 27 unique studies, were included in the review. Of these, 8 records were included in the narrative synthesis only, 10 were included in the meta‐analysis only and 10 were included in both.

Data requests were made to authors of studies lacking sex‐disaggregated outcomes, covering 247 reports across 154 corresponding authors. Contact could not be established for 16% of authors, often due to unavailable or undeliverable e‐mail addresses, particularly for older publications; 47% did not respond, 19% indicated that the data were no longer accessible, 4% declined to share data and 8% provided additional data (Figure 1).

All titles and abstracts were screened by two independent reviewers with overall agreement of 95.12%. Full‐text papers were obtained and independently screened for final inclusion, with an agreement rate of 80.44%. Studies excluded at the full‐text screening stage are detailed in Table S5.

### 2.3 Data Extraction

The following data were extracted: author, publication date, journal, country, publication type, funding source, reported conflict(s) of interest, study design, inclusion and exclusion criteria, study setting, use of ‘sex’ or ‘gender’, sample co‐morbidities, AUD assessment method, participant drinking status at baseline, alcohol‐related measures and outcomes, secondary measures and outcomes, number of data collection points, attrition, intervention details and alcohol use outcomes at baseline and follow‐up. Where data were only included in graphs/figures, values were estimated using Webplot Digitizer 3.1 (http://arohatgi.info/WebPlotDigitizer). See Table S2 for all required data conversions.

### 2.4 Risk of Bias

For outcomes derived from RCTs, the Cochrane Risk of Bias (RoB) 2 tool was employed to assess quality [35]. The JBI Critical Appraisal Tool was used for non‐randomised studies, evaluating criteria pertinent to cohort designs [36]. Each study was assessed independently by two reviewers using standardised templates. Once independent evaluations were complete, reviewers discussed any discrepancies (agreement rate: 73%). Industry sponsorship was identified in 48.28% of reports, primarily through medication donations (64%), unspecified support (21%), or direct monetary contributions (14%). Seven reports were flagged as high risk of bias due to disclosed conflicts of interest or insufficient transparency. High risk of bias, determined by RoB 2, the JBI, and conflicts of interest, was factored into analyses. GRADE assessments [37] were carried out for the three primary outcomes (binary results, between‐subject continuous outcomes [treatment vs. control] within‐subject continuous outcomes).

### 2.5 Narrative Synthesis

Narrative synthesis was conducted following Popay et al. [38]. The initial stage involved data visualisation (Figure S5). The visualised data facilitated a preliminary synthesis of the findings, helping to organise the results and identify patterns across studies regarding the direction and magnitude of effects while also beginning to explore influential factors. Descriptive summaries of each study were drafted within the categories of pharmacotherapy and mechanism of action.

### 2.6 Meta‐Analytic Approach

All meta‐analyses were conducted in R (version 2024.12.0) using the ‘metafor’, ‘dmetar’ and ‘dplyr’ packages. Multi‐level random‐effects models were used to account for the nested data structure, where multiple effect sizes were drawn from the same study. Outcomes were coded so that larger effect sizes consistently indicated a positive treatment response (e.g., greater abstinence or reduced consumption). Outcomes where higher values reflected poorer responses (e.g., relapse or greater drinking days) were reverse coded before analysis.

Between‐subject effects were calculated using Cohen's *d* as the effect size metric (standardised mean difference; SMD). Two between‐subject models were examined: one including all comparisons (e.g., treatment vs. another drug or control), and a restricted model including only comparisons against true control conditions (placebo, treatment as usual or no treatment) to enhance interpretability. Within‐subject effects were calculated as standardised mean change scores (SMCC) using raw score standardisation. A pre–post correlation of 0.7 was conservatively imputed for SMCC calculations. Binary outcomes (e.g., abstinence, relapse) were analysed using log‐transformed risk ratios (logRR). All models were conducted on the log scale, and final estimates were exponentiated for interpretability. Effect sizes were interpreted using conventional benchmarks: 0.2 (small), 0.5 (moderate), and 0.8 (large) for both SMD and SMCC. Risk ratios were interpreted on the log scale, with values of 1.0 indicating no effect.

Subgroup analyses and meta‐regression models were conducted to explore whether treatment effects differed by, or interacted with, treatment class, region, baseline drinking status, treatment duration (categorised as ≤ 12 weeks or > 12 weeks), presence of psychiatric comorbidities, and RoB. Continuous moderators included treatment duration, sample mean age, and baseline alcohol consumption. As pre‐specified in the PROSPERO protocol, subgroup analyses were conducted only where four or more independent trials were available, and meta‐regressions were conducted only where 10 or more independent trials were available [39].

Heterogeneity was assessed using *I* ^2^ statistics derived from multilevel variance components [40]. Publication bias was examined through funnel plots and Egger's regression test. As Egger's test cannot be applied to multi‐level models, single‐level models (rma.uni) were also conducted. Statistical power was evaluated for each effect size using the standard error derived from the reported sampling variance and the pooled effect size estimate obtained from the treatment vs. control meta‐analysis. Minimum per‐group sample sizes required to detect the overall pooled effect and the observed sex‐by‐treatment interaction were estimated using the ‘pwr.t.test’ function from the ‘pwr’ package. All primary analyses, including domain‐level models, subgroup, and moderator analyses were pre‐specified and registered on PROSPERO. Full analytic code is available in Supporting Information.

## 3 Results

### 3.1 Study Characteristics

The searches yielded 14,814 citations, of which 28 met inclusion criteria (Figure 1). Of 27 unique studies, a total of 25,041 participants were included; 6316 were female (25% of the sample) and 18,647 were male. Sample sizes varied between 12 and 14,697 participants, with a mean of 927 (±2984). The percentage of female participants in the trials ranged from 3% to 59%, with a mean of 32.11% (±12.44). Most reports primarily used only the term ‘sex’ (*n* = 12), while 8 used only ‘gender’; 6 reports employed both terms interchangeably, and 2 did not mention either. Only two studies defined how or explicitly addressed whether they were assessing sex differences.

Three reports employed non‐randomised designs [41, 42, 43] (including secondary analyses of health records and longitudinal studies), and three reports were open‐label trials [44, 45, 46]. The remaining 22 reports consisted of RCTs (including RCT‐derived data) [47, 48, 49, 50, 51, 52, 53, 54, 55, 56, 57, 58, 59, 60, 61, 62, 63, 64, 65, 66, 67, 68]. Naltrexone was the most frequently studied pharmacotherapy (*n* = 10) [45, 46, 48, 50, 53, 56, 58, 63, 65, 68], followed by baclofen (*n* = 6) [43, 44, 54, 55, 60, 64], varenicline (*n* = 3; two of which were the same trial) [49, 51, 62] and (es)citalopram (*n* = 3) [47, 67, 69]. Only one report each evaluated acamprosate [59], nalmefene [57], flupenthixol [66] and levetiracetam [52], while two studies compared multiple interventions [41, 42] (see Table 2).

**TABLE 2:** Study characteristics of included reports (ordered by pharmacotherapy and date).

Report	Treatment	Design	Population	Outcome	Total sample finding	Summary sex‐related findings
GABAergic/glutamatergic agents						
Buri et al. (2007)^c^ Switzerland	Acamprosate vs. disulfiram + residential program	12 months Naturalistic prospective multicentre study	AUD *n* = 76 37% female Participants drinking at baseline	Abstinence rate	—	Acamprosate (f): 3/8 abstinent. Disulfiram (f): 7/20 abstinent. Acamprosate (m): 4/8 abstinent Disulfiram (m): 13/40 abstinent.
				Drinks per drinking day	—	Acamprosate (f): 4.43 ± 6.43 (*n* = 7). Disulfiram (f): 7.43 ± 10.44 (*n* = 21). Acamprosate (m): 3.14 ± 4.98 (*n* = 7). Disulfiram (m): 6.06 ± 8.84 (*n* = 35).
Mason and Lehert (2012)^c^ Mixed	Acamprosate (1642–3000 mg) vs. placebo + counselling	2–12 months IPD of RCTs	AUD *n* = 6111 22% female Mixed baseline drinking status	Abstinent days	Acamprosate demonstrated significantly greater *Abstinent Days* compared to placebo (*p* < 0.001).	No significant interaction effect was found between treatment and sex on *Abstinent Days.* Acamprosate demonstrated significantly greater *Abstinent Days* to placebo for women and men (*p* < 0.001).
				Abstinence rate	Acamprosate demonstrated significantly greater *Abstinence Rate* compared to placebo (*p* < 0.001).	No significant interaction effect was found between treatment and sex on *Abstinence Rate*. Acamprosate demonstrated significantly greater *Abstinence Rate* to placebo for women and men (*p* < 0.001).
				No heavy drinking days	Acamprosate demonstrated significantly greater *No Heavy Drinking Days* compared to placebo (*p* < 0.001).	No significant interaction effect was found between treatment and sex on *No Heavy Drinking Days.* Acamprosate demonstrated significantly greater *No Heavy Drinking Days* to placebo for women and men (*p* < 0.001).
Witte et al. (2012)^c^ USA	Acamprosate (2000 mg) and Escitalopram (15.8 mg) vs. placebo and escitalopram (16.4 mg) + medical management	12 weeks Double‐blind placebo‐controlled RCT	AUD and MDD *n* = 23 43% female Participants drinking at baseline	Drinking days	Naltrexone significantly reduced *Drinking Days* (*p* < 0.0001).	No significant sex differences in reductions of *Drinking Days* (*p* = 0.94). Both men and women reduced *Drinking Days* (*p* < 0.0001).
Crits‐Cristoph et al. (2016)^c^ USA	Acamprosate vs. naltrexone (injectable) vs. naltrexone (oral) vs. TAU + psychosocial therapy	Secondary analysis of naturalistic data	AUD *n* = 14,697 25% female Participants drinking at baseline	Abstinence	Only naltrexone (injectable) participants were significantly more likely to achieve abstinence at discharge compared to TAU (*p* = 0.015, OR = 0.65).	Acamprosate (f): 17/26 abstinent. Naltrexone (injectable; f): 39/56 abstinent. Naltrexone (oral; f): 30/42 abstinent. TAU (f): 2629/3628 abstinent. Acamprosate (m): 22/34 abstinent. Naltrexone (injectable; m): 113/142 abstinent. Naltrexone (oral; m): 40/68 abstinent. TAU (m): 7970/10701 abstinent.
				Drinks per drinking day	—	Acamprosate (f): 2.46 ± 6.48 (*n* = 26). Naltrexone (injectable; f): 2.07 ± 5.65 (*n* = 56). Naltrexone (oral; f): 3.21 ± 8.17 (*n* = 42). TAU (f): 2.11 ± 5.97 (*n* = 3613). Acamprosate (m): 2.44 ± 6.44 (*n* = 34). Naltrexone (injectable; m): 1.55 ± 5.17 (*n* = 141). Naltrexone (oral; m): 2.03 ± 5.65 (*n* = 68). TAU (m): 1.94 ± 5.84 (*n* = 10,646).
Flannery et al. (2004)^c^ USA	Baclofen (30 mg daily) within‐subjects design + motivation enhancement therapy	12 weeks Open‐label trial	AUD *n* = 12 25% female Participants drinking at baseline	Drinks per drinking day	Significant decrease in *Drinks per Drinking Day* from baseline after 12 weeks of treatment (*p* < 0.01; −61.8%).	12 weeks (f): 2.73 ± 0.15 (*n* = 3). Baseline (f): 7.16 ± 2.99 (*n* = 3). 12 weeks (m): 7.19 ± 2.15 (*n* = 9). Baseline (m): 7.81 ± 1.99 (*n* = 9).
Garbutt et al. (2010)^b^ USA	Baclofen (30 mg) vs. placebo + BRENDA sessions	12 weeks Double‐blind placebo‐controlled RCT	AUD *n* = 80 45% female Participants abstinent at baseline	Heavy drinking days	Neither group demonstrated a significant reduction in *Heavy Drinking Days* (*p* = 0.56 for baclofen). The interaction between treatment group and time was also not significant (*p* = 0.73).	No moderating effect of sex on *Heavy Drinking Days* between treatment groups (*p* = 0.91).
				Abstinent days	Neither group demonstrated a significant reduction in *Abstinent Days* (*p* = 0.50 for baclofen). The interaction between treatment group and time was also not significant (*p* = 0.61).	No moderating effect of sex on *Abstinent Days* between treatment groups (*p* = 0.36).
de Beaupaire (2012)^c^ France	Baclofen (147 mg daily) within‐subjects design	24 months Longitudinal observational study	AUD *n* = 100 30% female Participants drinking at baseline	Drinks per day	—	24 months (f): 70.67 ± 79.3 (*n* = 30). Baseline (f): 183.67 ± 66.31 (*n* = 30). 24 months (m): 82.43 ± 119.5 (*n* = 70). Baseline (m): 224.86 ± 82.42 (*n* = 70).
Reynaud et al. (2017)^b^ France	Baclofen (180 mg) vs. placebo + BRENDA sessions	26 weeks Multicentre double‐blind placebo‐controlled RCT	AUD *n* = 310 27% female Participants abstinent at baseline	Abstinence rate	No significant differences in *Abstinence Rate* between Baclofen or placebo groups (*p* = 0.619).	Significant difference in *Abstinence Rate* between Baclofen and placebo in female participants (*p* = 0.032), greater abstinence in Baclofen.
Garbutt et al. (2021)^b^ USA	Baclofen (30 mg vs. 90 mg daily) vs. placebo + medical management	16 weeks Double‐blind placebo‐controlled RCT	AUD *n* = 120 47% female Participants drinking at baseline	Heavy drinking days	Baclofen (30 and 90 mg) groups demonstrated significant reductions in *Heavy Drinking Days* compared to placebo (*p* = 0.018, *d* = 0.51).	Significant moderation effect of sex on reduction of *Heavy Drinking Days* between treatment groups (*p* = 0.005).
				Heavy drinking days	Baclofen (90 mg) group demonstrated a significantly greater reduction in *Heavy Drinking Days* compared to placebo (*p* = 0.044, *d* = 0.39).	No significant difference in reduction of *Heavy Drinking Days* between Baclofen (90 mg) and placebo in men (*p* = 0.063, *d* = 0.36). Not significant in women (*p* = 0.33, *d* = 0.19).
				Heavy drinking days	—	Significant difference in reduction of *Heavy Drinking Days* between Baclofen (30 mg) and Placebo in women (*p* = 0.002, *d* = 0.61). Not significant in men (*p* = 0.69).
				Abstinent days	Baclofen (90 and 30 mg) groups demonstrated significant increases in *Abstinent Days* compared to placebo (*p* = 0.028, *d* = 0.49).	Significant moderation effect of sex on increase of *Abstinent Days* between treatment groups (*p* = 0.045).
				Abstinent days	Baclofen (90 mg) group demonstrated a significantly greater increase in *Abstinent Days* compared to placebo (*p* = 0.015, *d* = 0.47).	No significant difference in increase of *Abstinent Days* between Baclofen (90 mg) and placebo in men (*p* = 0.096, *d* = 0.32) and women (*p* = 0.063, *d* = 0.36).
				Abstinent days	—	Significant difference in increase of *Abstinent Days* between Baclofen (30 mg) and placebo in women (*p* = 0.007, *d* = 0.52). Not significant in men (*p* = 0.61).
Morley et al. (2022)^a^ Australia	Baclofen (30–75 mg daily) vs. placebo	12 weeks Post hoc analysis of double‐blind placebo‐controlled RCT	AUD *n* = 104 29% female Participants abstinent at baseline	Abstinent days	Baclofen (30 and 75 mg) demonstrated significantly greater *Abstinent Days* compared to placebo (*p* < 0.01).	Significant differences in the increase of *Abstinent Days* between Baclofen and placebo in women (*p* = 0.046), greater *Abstinent Days* in Baclofen. Not significant in men.
Fertig et al. (2012)^b^ USA	Levetiracetam (2000 mg daily) vs. placebo + BBCET sessions	16 weeks Multicentre double‐blind placebo‐controlled RCT	AUD *n* = 130 24% female Participants drinking at baseline	Heavy drinking days	Neither group demonstrated significant reductions in *Heavy Drinking Days*; the interaction between study week and treatment group was non‐significant (*p* > 0.19).	No significant interaction effect between treatment and sex on *Heavy Drinking Days* (*p* = 0.41).
Opioid receptor agonists						
Hashimoto et al. (2022)^b^ Japan	Nalmefene (10 or 20 mg daily) vs. placebo + BRENDA sessions	24 weeks Post hoc analysis of multicentre double‐blind placebo‐controlled RCT	AUD *n* = 666 31% female Participants drinking at baseline	Heavy drinking days	Nalmefene (10 and 20 mg) demonstrated significant reductions in *Heavy Drinking Days* compared to placebo at 12 weeks (*p* < 0.001).	No significant interaction effect between treatment and sex on *Heavy Drinking Days* (*p* = 0.678).
				Heavy drinking days	Nalmefene (10 and 20 mg) demonstrated significant reductions in *Heavy Drinking Days* compared to placebo at 24 weeks (*p* < 0.001).	No significant interaction effect between treatment and sex on *Heavy Drinking Days* (*p* = 0.618).
Krystal et al. (2001)^c^ USA	Naltrexone (50 mg daily) within‐subjects design + 12‐step facilitation	12 months Multicentre double‐blind placebo‐controlled RCT	AUD veterans *n* = 209 3% female Participants abstinent at baseline	Drinking days	No significant differences between naltrexone and placebo in *Drinking Days* following 12 weeks of treatment (95% CI −7.7 to 1.9).	12 months (f): 10.13 ± 21.87 (*n* = 6). Baseline (f): 75.26 ± 18.97 (*n* = 6). 12 months (m): 15.25 ± 23.4 (*n* = 203). Baseline (m): 65.63 ± 30.15 (*n* = 203).
				Drinks per drinking day	No significant differences between naltrexone and placebo in *Drinks per Drinking Day* following 12 weeks of treatment (95% CI −1.8 to 2.4).	12 months (f): 6.54 ± 3.46 (*n* = 6). Baseline (f): 7.67 ± 1.89 (*n* = 6). 12 months (m): 9.68 ± 10.31 (*n* = 203). Baseline (m): 13.21 ± 7.66 (*n* = 203).
Garbutt et al. (2005)^a^ USA	Naltrexone (190 mg vs. 380 mg monthly injections) vs. placebo + BRENDA sessions	24 weeks Multicentre double‐blind placebo‐controlled RCT	AUD *n* = 624 32% female Participants drinking at baseline	Heavy drinking days	—	Significant interaction between treatment (380 mg and 190 mg) and sex on *Heavy Drinking Days* (*p* = 0.002).
				Heavy drinking days	Naltrexone (380 mg) group demonstrated significantly lower *Heavy Drinking Days* compared to placebo group (*p* = 0.03).	Significant differences in reduction of *Heavy Drinking Days* between NTX (380 mg) and placebo in men (HR = 0.56, *p* < 0.001), greater reduction in NTX (380 mg).
Pettinati et al. (2008)^a^ USA	Naltrexone (150 mg daily) vs. placebo + individual CBT	12 weeks Double‐blind placebo‐controlled RCT	AUD and cocaine use disorder *n* = 164 29% female Participants abstinent at baseline	Abstinence rate	—	Significant interaction effect between treatment and sex on rates of *Abstinence Rate* (*p* = 0.02). No significant difference between Naltrexone and placebo on rates of *Abstinence Rate* in women (*p* = 0.18) or men (*p* < 0.09).
Baros et al. (2008)^a^ USA	Naltrexone (50 daily) vs. placebo + individual CBT	12 weeks Pooled analysis of two double‐blind placebo‐controlled RCTs	AUD *n* = 211 27% female Participants abstinent at baseline	Abstinent days	—	Significant difference in increase of *Abstinent Days* between NTX and placebo in men (*p* = 0.03, *d* = 0.36), greater increase *Abstinent Days* in NTX. Not significant in women.
				Heavy drinking days	—	Significant difference in reduction of *Heavy Drinking Days* between NTX and placebo in men (*p* = 0.03, d = 0.36), greater reduction in NTX. Not significant in women.
				Total drinks	—	Significant difference in reduction of *Total Drinks* between NTX and placebo in men (*p* = 0.04, *d* = 0.36), greater reduction in NTX. Not significant in women.
Greenfield et al. (2010)^b^ USA	Naltrexone (100 mg daily) vs. placebo + medical management	16 weeks Post hoc analysis of multicentre double‐blind placebo‐controlled RCT	AUD *n* = 307 32% female Participants abstinent at baseline	Abstinent days	Both groups demonstrated increases. No significant differences between the groups (*p* = 0.25).	No significant difference in increase of *Abstinent Days* between Naltrexone and placebo in men (*p* = 0.06) and women (*p* = 0.092).
Stoner et al. (2015)^c^ USA	Naltrexone (50 mg daily) within‐subjects	8 weeks RCT	AUD *n* = 37 49% female Participants abstinent at baseline	Drinking days	—	8 weeks (f): 52.6 ± 39.06 (*n* = 18). Baseline (f): 73.72 ± 27.66 (*n* = 18). 8 weeks (m): 54 ± 41.51 (*n* = 19). Baseline (m): 71.95 ± 23.33 (*n* = 19).
				Drinks per drinking day	—	8 weeks (f): 2.41 ± 0.83 (*n* = 18). Baseline (f): 8.33 ± 4.28 (*n* = 18). 8 weeks (m): 6.03 ± 3.91 (*n* = 19). Baseline (m): 9.68 ± 4.59 (*n* = 19).
Yoon et al. (2016)^a^ USA	Naltrexone (150 mg daily) within‐subjects	8 weeks Open‐label trial	AUD *n* = 24 54% female Participants drinking at baseline	Drinking days	Naltrexone significantly reduced *Drinking Days* (*p* < 0.0001).	No significant sex differences in reductions of *Drinking Days* (*p* = 0.18). Both men and women *Drinking Days* pDD (*p* < 0.0001).
				Drinks per drinking day	Naltrexone significantly reduced *Drinks per Drinking Day* (*p* < 0.0001).	No significant sex differences in reductions of *Drinks per Drinking Day* (*p* = 0.18). Both men and women reduced *Drinks per Drinking Day* (*p* < 0.0001).
Korthuis et al. (2017)^c^ USA and Canada	Naltrexone (injectable; 380 mg daily) vs. TAU and within‐subjects + medical management and referral to counselling	16 weeks Open‐label, randomised pilot trial	AUD moderate/severe and HIV *n* = 23 35% female Participants drinking at baseline	Drinking days	—	16 weeks Naltrexone (f): 6.83 ± 4.02 (*n* = 6). Baseline Naltrexone (f): 17.83 ± 8.86 (*n* = 6). 16 weeks Naltrexone (m): 5.17 ± 7 (*n* = 6). Baseline Naltrexone (m): 11.33 ± 9.44 (*n* = 6).
				Drinking days	—	Naltrexone (f): 6.83 ± 4.02 (*n* = 6). TAU (f): 14 ± 19.8 (*n* = 2). Naltrexone (m): 5.17 ± 7 (*n* = 6). TAU (m): 9.11 ± 10.03 (*n* = 9).
				Drinks per drinking day	—	16 weeks Naltrexone (f): 2.58 ± 2.1 (*n* = 6). Baseline Naltrexone (f): 7.76 ± 5.25 (*n* = 6). 16 weeks Naltrexone (m): 3.37 ± 4.7 (*n* = 6). Baseline Naltrexone (m): 7.22 ± 5.41 (*n* = 6).
				Drinks per drinking day	—	Naltrexone (f): 2.58 ± 2.1 (*n* = 6). TAU (f): 0.05 ± 0.08 (*n* = 2). Naltrexone (m): 3.37 ± 4.7 (*n* = 6). TAU (m): 5.2 ± 4.24 (*n* = 9).
Goldstein (2023)^a^ USA	Naltrexone (injectable; 380 mg monthly) vs. placebo + harm reduction counselling	12 weeks Post hoc analysis of double‐blind placebo‐controlled RCT	AUD and homeless *n* = 152 14% female Participants drinking at baseline	Drinks per drinking day	Both groups demonstrated reductions. No significant differences between the groups (linear *p* = 0.634).	No moderating effect of sex on *Drinks per Drinking Day* between treatment groups (*p* = 0.06).
				Drinking days	Both groups demonstrated reductions. No significant differences between the groups (linear *p* = 0.458).	No moderating effect of sex on *Drinking Days* between treatment groups (*p* = 0.52).
Davis et al. (2023)^c^ USA	Naltrexone (injectable; 380 mg monthly) and Buprenorphine (2 mg daily) vs. placebo + medical management	12 weeks Multicentre double‐blind placebo‐controlled RCT	AUD *n* = 50 16% female Participants drinking at baseline	Heavy drinking days	No significant interaction between treatment group (between naltrexone/buprenorphine and placebo) and time (baseline to week 12) for *Heavy Drinking Days* (*p* = 0.09).	Naltrexone (f): 9.2 16.2 (*n* = 4). Placebo (f): 49.1 ± 56.7 (*n* = 4). Naltrexone (m): 25.5 ± 30.8 (*n* = 19). Placebo (m): 18.3 ± 22.2 (*n* = 23).
				Abstinent days	No significant interaction between treatment group (between naltrexone/buprenorphine and placebo) and time (baseline to week 8) for *Abstinent Days* (*p* = 0.89).	Naltrexone (f): 9.2 ± 16.2 (*n* = 4). Placebo (f): 71.4 ± 47.8 (*n* = 4). Naltrexone (m): 47.2 ± 38.6 (*n* = 19). Placebo (m): 35.3 ± 29.1 (*n* = 23).
				Drinks per drinking day	No significant interaction between treatment group (between naltrexone/buprenorphine and placebo) and time (baseline to week 8) for *Drinks per Drinking Day* (*p* = 0.08).	Naltrexone (f): 0.8 ± 1.4 (*n* = 4). Placebo (f): 3.1 ± 2.7 (*n* = 4). Naltrexone (m): 3.1 ± 4.1 (*n* = 19). Placebo (m): 1.7 ± 1.4 (*n* = 23).
Serotonergic/dopaminergic agents						
Naranjo et al. (2000)^a^ Canada	Citalopram (40 mg daily) vs. placebo + prescriptive advice, pamphlets, progress monitoring	12 weeks Post hoc analysis of double‐blind placebo‐controlled RCT	AUD mild/moderate *n* = 61 44% female Participants drinking at baseline	Drinks per day	Both groups demonstrated reductions (*p* < 0.01). No significant differences between the groups.	Significant interaction effect was found between treatment and sex on *Drinks per Day* with depression or anxiety as covariates (*p* < 0.05). Men receiving citalopram demonstrated significantly greater reductions in *Drinks per Day* than women receiving citalopram (*p* < 0.05).
			
				Drinks per drinking day	Both groups demonstrated reductions (*p* < 0.01). No significant differences between the groups.	No significant interaction effect was found between treatment and sex on *Drinks per Drinking Day* with any covariates (*p* > 0.086).
Abstinent days	Both groups demonstrated reductions (*p* < 0.01). No significant differences between the groups.	No significant interaction effect was found between treatment and sex on *Abstinent Days* with any covariates (*p* > 0.75).
Adamson et al. (2015)^a^ New Zealand	Citalopram (40 mg daily) and Naltrexone (50 mg daily) vs. placebo and Naltrexone (50 mg daily) + clinical case management with addiction clinicians	12 weeks Double‐blind placebo‐controlled RCT	AUD and MDD *n* = 138 59% female Participants drinking at baseline	Abstinent days	Both Citalopram and placebo groups demonstrated significant reductions. No significant differences between the groups (*p* = 0.104).	No significant interaction effect between treatment and sex on *Abstinent Days* (*p* = 0.051).
				Drinks per drinking day	—	Significant difference in reduction of *Drinks per Drinking Day* between NTX and placebo in men (*p* = 0.05, *d* = 0.36), greater reduction in NTX. Not significant in women.
Wiesbeck et al. (2003)^a^ Germany and Austria	Flupenthixol (injectable; 10 mg fortnightly) vs. placebo + individual or group psychotherapy; AA advised	6 months Post hoc analysis of multicentre double‐blind placebo‐controlled RCT	AUD moderate/severe *n* = 281 27% female Participants abstinent at baseline	Relapse rate	Flupenthixol group demonstrated significantly greater *Relapse Rates* compared to placebo (*p* < 0.01).	Significant difference in *Relapse Rates* between Flupenthixol and placebo for male participants (*p* < 0.0001), greater *Relapse Rates* in treatment group. No significant differences in female participants (*p* = 0.473).
Falk et al. (2015)^b^ USA	Varenicline (2 mg daily) vs. placebo + self‐help video modules	13 weeks Post hoc analysis of multicentre double‐blind placebo‐controlled RCT	AUD *n* = 200 30% female Participants drinking at baseline	Heavy drinking days	Varenicline group demonstrated significantly lower *Heavy Drinking Days* compared to placebo group (*p* = 0.03; *d* = 0.31).	No moderating effect of sex on treatment efficacy (*p* > 0.05).
				Drinks per day	Varenicline group demonstrated significantly fewer *Drinks per Day* compared to placebo group (*p* = 0.03; *d* = 0.29).	No moderating effect of sex on treatment efficacy (*p* > 0.05).
				Drinks per drinking day	Varenicline group demonstrated significantly fewer *Drinks per Drinking Day* compared to placebo group (*p* = 0.03; *d* = 0.26).	No moderating effect of sex on treatment efficacy (*p* > 0.05).
O'Malley et al. (2018)^a^ USA	Varenicline (2 mg daily) vs. placebo + medical management	16 weeks Multicentre double‐blind placebo‐controlled RCT	AUD smokers *n* = 131 30% female Participants drinking at baseline	Heavy drinking days	No significant differences in the reduction of *Heavy Drinking Days* between Varenicline and placebo groups (*p* = 0.80).	Significant interaction between treatment and sex on *Heavy Drinking Days* (*p* = 0.03); no significant difference in reduction of *Heavy Drinking Days* between Varenicline and placebo in men (*p* = 0.09, *d* = 0.45). No significant difference in *Heavy Drinking Days* reduction between Varenicline and placebo in women (*p* = 0.15, *d* = −0.53).
				No heavy drinking days rates	—	Significant difference *No Heavy Drinking Days Rates* between Varenicline and placebo in men (CI 0.22–1.03), greater *No Heavy Drinking Days Rates* in Varenicline. No significant differences in women (CI −1.21 to 0.04).
Bold et al. (2019)^b^ USA Follow‐up data of O'Malley et al. (2018)	Varenicline (2 mg daily) vs. Placebo + medical management	16 weeks Multicentre double‐blind placebo‐controlled RCT	AUD smokers *n* = 131 30% female Participants drinking at baseline	Heavy drinking days	No significant differences in the reduction of *Heavy Drinking Days* between Varenicline and placebo groups at 12‐month follow‐up (*p* = 0.89).	No significant interaction effect between treatment and sex on *Heavy Drinking Days* at 12‐month follow‐up (*p* = 0.32).
				No heavy drinking days rates	No significant differences in *No Heavy Drinking Days Rates* between Varenicline and placebo groups at 12‐month follow‐up (*p* > 0.13).	Significant interaction between treatment and sex on *No Heavy Drinking Days Rates* at 12‐month follow‐up (*p* = 0.01); Varenicline group demonstrated significantly greater *No Heavy Drinking Days Rates* compared to placebo in men (*p* = 0.03). This was not significant in women (*p* > 0.11).

*Note:* Studies are ordered by pharmacotherapy class and publication year. Doses refer to oral tablet or capsule administration unless otherwise indicated. Where dosage information was not reported, data were missing. Where additional psychosocial support was not described, none was administered. Findings are summarised as presented in the original reports; statistical significance reflects authors’ analyses. For studies not included in the meta‐analysis, sex‐disaggregated means and standard deviations are reported as supplied by authors or computed from shared datasets (*n*, *M* ± SD for continuous outcomes, and event counts for binary outcomes).

Abbreviations: AA, Alcoholics Anonymous; AUD, alcohol use disorder; CI, confidence interval; IPD, individual participant data; MDD, major depressive disorder; OR, odds ratio; RCT, randomised controlled trial; TAU, treatment as usual.

^a^ Reports included in systematic review and meta‐analysis.

^b^ Reports included in systematic review only.

^c^ Reports included in meta‐analysis only (sex‐related findings are data input in meta‐analyses).

Binary outcomes included *Abstinence Rate*, *Relapse Rate* and *No Heavy Drinking Days Rate*. Continuous outcomes included both consumption frequency related measures: Count/Percent/Event Rate of *Drinking Days*, *Heavy Drinking Days*, *Abstinent Days* and *Non‐Heavy Drinking Days*; and consumption quantity related measures: *Drinks per Drinking Day*, *Drinks per Day* and *Total Drinks*.

### 3.2 Risk of Bias

Forty‐seven outcomes across 22 RCTs were assessed for risk of bias (see Figure S1). Overall, 55.3% of outcomes were rated as having some concerns, with the remaining 44.7% rated as high risk of bias. Outcome measurement was the most robust domain (95.7% low risk), reflecting consistent use of validated tools and appropriate blinding. The most problematic domain was the selection of reported results (91.5% some concern/high risk), stemming from post hoc analyses where sex/gender was explored without clearly pre‐specified outcomes. Five non‐RCTs were assessed using the JBI checklist and scores ranged from moderate to high, with individual scores between 54.6% and 81.8%. All studies measured exposures consistently across groups and reported sufficient follow‐up periods. However, only 40% adequately addressed attrition and described attrition‐management strategies.

### 3.3 Meta‐Analysis

#### 3.3.1 Primary Analyses

There were small but positive treatment effects for overall efficacy measures, see Table 3 for full results. Positive effect sizes reflect beneficial treatment responses (i.e., reduced consumption or increased abstinence). Male participants were the reference group in all sex‐moderator analyses; positive moderator estimates for continuous outcomes or ratios of relative risks ≥ 1 for binary outcomes indicate greater treatment effects in male participants.

**TABLE 3:** Main treatment effect, pooled estimates by sex and sex moderation effect by multi‐level meta‐analysis of pharmacotherapy effectiveness.

	*k*/*N* trials	*I* ^2^	Main treatment effect					Male estimate		Female estimate		Sex moderation effect			
			*N* (*n*_tx_)	ES (SE)	95% CI		*p*	*N* (*n*_tx_)	ES (SE)	*N* (*n*_tx_)	ES (SE)	ES (SE)	95% CI		*p*
					LB	UB							LB	UB	
Binary outcomes															
Abstinence or no heavy drinking days	9/4	94.79	15,194 (590)	0.96 (0.31)	0.52	1.74	0.879	11,298 (404)	0.97 (0.31)	3896 (186)	0.93 (0.31)	1.04 (0.09)	0.87	1.25	0.663
Continuous outcomes (consumption reduction)															
Treatment vs. control	31/13	88.84	22,394 (4534)	0.23 (0.11)	0.01	0.45	0.042	16,813 (3422)	0.25 (0.13)	5581 (1112)	0.18 (0.08)	0.05 (0.04)	−0.02	0.12	0.174
Treatment vs. other drug or control	36/14	88.85	22,464 (4548)	0.22 (0.11)	0.01	0.43	0.042	16,855 (3429)	0.24 (0.11)	5609 (1119)	0.19 (0.12)	0.05 (0.07)	−0.09	0.18	0.505
Within‐subject change	30/15	96.62	1621	0.97 (0.22)	0.54	1.39	< 0.001	1151	0.92 (0.26)	470	1.00 (0.27)	−0.10 (0.30)	−0.70	0.50	0.740
Frequency outcomes only															
Treatment vs. control	21/13	93.60	22,310 (4491)	0.24 (0.13)	−0.02	0.50	0.065	16,766 (3398)	0.23 (0.16)	5544 (1093)	0.18 (0.08)	0.05 (0.04)	−0.02	0.13	0.165
Treatment vs. other drug or control	25/13	93.38	22,310 (4491)	0.23 (0.13)	−0.04	0.48	0.091	16,766 (3398)	0.24 (0.14)	5544 (1093)	0.19 (0.14)	0.05 (0.08)	−0.11	0.20	0.574
Within‐subject change	17/12	98.37	1415	0.50 (0.54)	−0.55	1.55	0.348	998	0.51 (0.54)	417	0.47 (0.54)	0.02 (0.13)	−0.24	0.29	0.863
Quantity outcomes only															
Treatment vs. control	10/9	55.34	902 (488)	0.24 (0.14)	−0.03	0.51	0.078	615 (332)	0.27 (0.16)	287 (156)	0.24 (0.16)	0.08 (0.21)	−0.33	0.49	0.701
Treatment vs. other drug or control	11/10	60.04	972 (502)	0.25 (0.13)	0.01	0.49	0.047	657 (339)	0.28 (0.15)	315 (163)	0.21 (0.17)	0.07 (0.19)	−0.30	0.44	0.713
Within‐subject change	13/13	83.11	839	1.14 (0.16)	0.83	1.45	< 0.001	628	1.10 (0.16)	211	1.24 (0.18)	−0.15 (0.12)	−0.38	0.09	0.229

*Note:* This table presents the results of multi‐level mixed‐effects meta‐analyses examining pharmacotherapy effectiveness and the moderating effect of sex. The column main treatment effect reports the estimated pooled effect size from a model that did not include sex as a moderator. The columns under male and female are pooled subgroup estimates derived from a separate model that included sex as a moderator. For this sex moderation effect, female participants constitute the reference group (intercept) and male participants are represented by the coefficient. *k/N* = the number of effect sizes (outcomes) and the number of independent trials (studies) contributing to the analysis. *I* ^2^ = the total heterogeneity across the effect sizes (in percentage). *N* (*n*_tx_) = the total sample size (*N*) for that analysis (or sex subgroup), with the number of participants receiving the active treatment (*n*_tx_) provided in parentheses. ES (SE) = the estimated pooled effect size (ES) with the standard error (SE) reported in parentheses. 95% CI = 95% confidence intervals with the lower bound (LB) and upper bound (UB), indicating the range of values within which the true effect lies with 95% certainty. *p* = *p* value associated with the test of the null hypothesis that the effect is zero. All *p* values are reported to three decimal places. The specific effect measure (ES) and its interpretation vary by both the o*utcome* (*binary or continuous*) and *model type* (*main effect or sex moderation effect*). For the main treatment effect and subgroup estimates (male/female): 1. The ES for binary outcomes is the exponentiated risk ratio (RR), where values ≥ 1 indicate a beneficial treatment effect (e.g., achieving abstinence). 2. For Continuous outcomes comparing treatment groups, the ES is the standardised mean difference (SMD). For within‐subject change (comparing pre‐ to post‐treatment), the ES is the standardised mean change coefficient (SMCC). For both SMD and SMCC, a positive ES indicates a beneficial treatment effect (i.e., greater consumption reduction). For the sex moderation effect: 1. The ES for binary outcomes is the ratio of risk ratios, where values ≥ 1 indicate greater treatment effect in male participants compared to female participants. 2. For continuous outcomes, the ES is the difference (Δ) in SMD between male and female participants. For within‐subject change, the ES is the difference (Δ) in the SMCC between male and female participants. For both ΔSMD and ΔSMCC, positive ES values indicate greater treatment response in male participants.

Inspection of forest plots revealed that all confidence intervals (CI) overlapped, so no observations were removed. Heterogeneity was high, with *I* ^2^ ranging from 55.34% to 98.37%. See Figures [Link], [Link] for forest plots.

For binary outcomes (*Abstinence* and *No Heavy Drinking Days Rate*), the pooled effect comparing treatment to control suggested minimal difference (RR = 0.96, *p* = 0.88, 95% CI [0.52, 1.74]), with wide confidence intervals encompassing both potential benefit and harm. Sex‐moderator analyses showed similarly imprecise effects, with no meaningful difference between male and female participants (ratio of risk ratio = 1.04, *p* = 0.66, 95% CI [0.87, 1.25]).

For continuous outcomes (consumption reduction), the pooled effects indicated small beneficial effects: treatment versus control (SMD = 0.23, *p* = 0.043, 95% CI [0.01, 0.45]), treatment to other drug or control (SMD = 0.22, *p* = 0.043, 95% CI [0.01, 0.43]), as well as within‐subject change (SMCC = 0.97, *p* < 0.001, 95% CI [0.54, 1.39]). Sex‐moderator effects were small and imprecise, with confidence intervals including zero: ΔSMD = 0.05, *p* = 0.51, 95% CI [−0.09, 0.18], treatment versus control; ΔSMD = 0.05, *p* = 0.17, 95% CI [−0.02, 0.12], treatment versus other drug or control; ΔSMCC = −0.10, *p* = 0.74, 95% CI [−0.70, 0.50], within‐subject change.

For frequency reduction, the pooled estimated and wide confidence intervals suggested little difference between treatment and control (SMD = 0.23, *p* = 0.091, 95% CI [−0.04, 0.48]). When adding sex as a moderator, there was no meaningful difference between male and female participants (ΔSMD = 0.05, *p* = 0.57, 95% CI [−0.11, 0.20]). Similar patterns were observed when comparing treatment to other drug or control and within‐subject change.

For quantity reduction, the pooled effect comparing treatment to other drug or control was small (SMD = 0.24, *p* = 0.078, 95% CI [−0.03, 0.51]), indicating that the true effect could range from minimal harm to moderate benefit. However, when excluding other drugs in the comparison, the pooled effect remained small, but the confidence interval excluded zero (SMD = 0.25, *p* = 0.047, 95% CI [0.01, 0.49]), suggesting a small to moderate beneficial effect. Neither of these comparisons was moderated by sex (ΔSMD = 0.08, *p* = 0.70, 95% CI [−0.33, 0.49], treatment vs. other drug or control; ΔSMD = 0.07, *p* = 0.71, 95% CI [−0.30, 0.44], treatment vs. control). Within‐subject analyses showed a positive treatment effect (SMCC = 1.14, *p* < 0.001, 95% CI [0.83, 1.45]), with sex‐moderator estimates close to zero (ΔSMCC = −0.15, *p* = 0.23, 95% CI [−0.38, 0.09]).

#### 3.3.2 Subgroup Analyses

In line with the registered protocol, a minimum of four studies was required to conduct categorical subgroup analyses, see Table 4 for full results.

**TABLE 4:** Subgroup main treatment effect, pooled estimates by sex and sex moderation effect by multi‐level meta‐analyses.

	*k*/*N* trials	*I* ^2^	Main treatment effect					Male estimate		Female estimate		Sex moderation effect			
			*N* (*n*_tx_)	SMD (SE)	95% CI		*p*	*N* (*n*_tx_)	SMD (SE)	*N* (*n*_tx_)	SMD (SE)	ΔSMD (SE)	95% CI		*p*
					LB	UB							LB	UB	
Opioid receptor agonists															
Overall efficacy	17/7	31.96	15,952 (1001)	0.17 (0.07)	0.04	0.30	0.001	11,836 (695)	0.20 (0.07)	4116 (306)	0.10 (0.09)	0.09 (0.08)	−0.06	0.24	0.236
Frequency outcomes	12/7	37.63	15,952 (1001)	0.18 (0.07)	0.05	0.31	0.001	11,836 (695)	0.23 (0.07)	4116 (306)	0.07 (0.09)	0.15 (0.08)	−0.01	0.31	0.067
Quantity outcomes	5/5	00.00	553 (279)	0.20 (0.09)	0.03	0.37	0.020	415 (206)	0.13 (0.10)	138 (73)	0.43 (0.18)	−0.31 (0.20)	−0.70	0.09	0.126
GABAergic/glutamatergic agents															
Overall efficacy	8/4	99.28	20,580 (3366)	0.43 (0.40)	−0.37	1.22	0.292	15,584 (2638)	0.49 (0.45)	4996 (728)	0.37 (0.46)	0.12 (0.41)	−0.68	0.93	0.762
Frequency outcomes	6/4	99.63	20,580 (3366)	0.39 (0.51)	−0.60	1.38	0.441	15,584 (2638)	0.42 (0.58)	15,584 (2638)	0.38 (0.59)	0.04 (0.56)	−1.06	1.14	0.943
Quantity outcomes	2/2	—	—	—	—	—	—	—	—	—	—	—	—	—	—
Sample drinking at baseline															
Overall efficacy	20/9	27.49	15,781 (1051)	0.08 (0.06)	−0.04	0.20	0.202	11,652 (690)	0.10 (0.09)	4129 (361)	0.04 (0.10)	0.09 (0.08)	−0.07	0.25	0.252
Frequency outcomes	13/9	40.16	15,781 (1051)	0.09 (0.08)	−0.06	0.24	0.225	11,652 (690)	0.08 (0.10)	4129 (361)	0.09 (0.10)	0.08 (0.09)	−0.10	0.25	0.393
Quantity outcomes	7/6	00.00	84 (43)	0.03 (0.09)	−0.15	0.22	0.716	47 (24)	0.09 (0.14)	37 (19)	−0.06 (0.21)	0.17 (0.21)	−0.24	0.57	0.424
Treatment length ≤ 12 weeks															
Overall efficacy	21/8	80.89	879 (476)	0.26 (0.18)	−0.10	0.62	0.150	754 (404)	0.26 (0.22)	336 (179)	0.30 (0.19)	0.05 (0.15)	−0.25	0.34	0.769
Frequency outcomes	12/8	88.96	879 (476)	0.29 (0.24)	−0.18	0.75	0.230	754 (404)	0.24 (0.28)	336 (179)	0.42 (0.15)	−0.04 (0.29)	−0.61	0.54	0.898
Quantity outcomes	9/8	63.34	879 (476)	0.26 (0.15)	−0.03	0.55	0.074	754 (404)	0.26 (0.19)	336 (179)	0.28 (0.18)	0.03 (0.21)	−0.38	0.44	0.890
Conducted in North America															
Overall efficacy	24/10	29.14	16,018 (1168)	0.12 (0.06)	0.00	0.24	0.040	11,866 (798)	0.16 (0.07)	4152 (370)	0.06 (0.09)	0.12 (0.07)	−0.01	0.25	0.080
Frequency outcomes	16/10	36.42	16,018 (1168)	0.13 (0.07)	−0.01	0.26	0.058	11,866 (798)	0.16 (0.08)	4152 (370)	0.03 (0.07)	0.15 (0.07)	0.01	0.23	0.049
Quantity outcomes	8/7	00.00	637 (322)	0.17 (0.08)	0.02	0.32	0.030	462 (230)	0.16 (0.11)	175 (92)	0.16 (0.23)	0.01 (0.19)	−0.36	0.37	0.981
High risk of bias															
Overall efficacy	5/14	91.98	15,048 (611)	0.29 (0.30)	−0.31	0.89	0.342	11,187 (419)	0.29 (0.30)	3861 (192)	0.20 (0.28)	0.21 (0.18)	−0.13	0.55	0.231
Frequency outcomes	5/9	96.00	15,048 (611)	0.23 (0.39)	−0.54	1.00	0.558	11,187 (419)	0.24 (0.45)	3861 (192)	0.23 (0.39)	0.11 (0.30)	−0.47	0.69	0.719
Quantity outcomes	4/5	—		—	—	—	—	—	—	—	—	—	—	—	—
Low/medium risk of bias															
Overall efficacy	7/15	20.89	1444 (723)	0.18 (0.05)	0.08	0.29	0.001	975 (481)	0.16 (0.10)	469 (242)	0.16 (0.08)	0.03 (0.11)	−0.19	0.25	0.794
Frequency outcomes	7/10	26.47	1444 (723)	0.21 (0.06)	0.09	0.33	0.001	975 (481)	0.23 (0.10)	259 (136)	0.16 (0.12)	0.11 (0.13)	−0.14	0.36	0.381
Quantity outcomes	5/5	00.00	480 (244)	0.11 (0.09)	−0.08	0.29	0.254	317 (156)	0.04 (0.11)	163 (88)	0.23 (0.16)	−0.19 (0.20)	−0.60	0.19	0.325
Sample comorbidity (*Y*)															
Overall efficacy	12/5	17.41	398 (201)	0.12 (0.08)	−0.04	0.27	0.133	242 (119)	0.00 (0.16)	156 (82)	0.23 (0.13)	−0.18 (0.16)	−0.50	0.14	0.267
Frequency outcomes	7/5	31.51	398 (201)	0.12 (0.11)	−0.09	0.34	0.260	242 (119)	0.01 (0.20)	156 (82)	0.28 (0.25)	−0.15 (0.24)	−0.63	0.32	0.529
Quantity outcomes	5/5	00.00	398 (201)	0.10 (0.10)	−0.10	0.30	0.344	242 (119)	−0.01 (0.13)	156 (82)	0.26 (0.16)	−0.27 (0.21)	−0.68	0.15	0.207
Sample comorbidity (*N*)															
Overall efficacy	16/8	94.41	21,996 (4333)	0.32 (0.16)	−0.00	0.64	0.051	16,571 (3303)	0.34 (0.16)	5425 (1030)	0.27 (0.17)	0.07 (0.04)	−0.01	0.15	0.096
Frequency outcomes	11/8	96.36	21,996 (4333)	0.33 (0.18)	−0.03	0.68	0.073	16,571 (3303)	0.34 (0.18)	5425 (1030)	0.29 (0.18)	0.06 (0.04)	−0.02	0.13	0.152
Quantity outcomes	5/4	75.96	504 (287)	0.41 (0.26)	−0.10	0.91	0.117	373 (213)	0.54 (0.28)	131 (74)	0.19 (0.31)	0.35 (0.27)	−0.18	0.87	0.194

*Note:* This table presents the results of subgroup multi‐level mixed‐effects meta‐analyses examining pharmacotherapy effectiveness (treatment vs. control) and the moderating effect of sex on continuous consumption‐reduction outcomes. The columns under main treatment effect report the estimated pooled standardised mean difference (SMD) from models without the sex moderator. The columns under male and female present pooled subgroup estimates derived from models including sex as a moderator. For the sex moderation effect, female participants constitute the reference group (intercept), and male participants are represented by the moderator coefficient. *k*/*N* = the number of effect sizes (outcomes) and the number of independent trials (studies) contributing to the analysis. *I* ^2^ = the total heterogeneity across the effect sizes (in percentage). *N* (*n*_tx_) = the total sample size (*N*) for that analysis (or sex subgroup), with the number of participants receiving the active treatment (*n*_tx_) provided in parentheses. SMD (SE) = the estimated pooled standardised mean difference (SMD) with the standard error (SE) reported in parentheses. ΔSMD = the difference between the SMD (of male and female participants). 95% CI = 95% confidence intervals with the lower bound (LB) and upper bound (UB), indicating the range of values within which the true effect lies with 95% certainty. *p* = *p* value associated with the test of the null hypothesis that the effect is zero. A positive SMD indicates a beneficial treatment effect (i.e., greater reduction in alcohol consumption). For the Sex Moderation Effect, a positive ΔSMD value indicates greater treatment response among male participants. Subgroups were only analysed when at least four independent trials contributed to the comparison, in accordance with the preregistered protocol: Opioid receptor agonists include primarily naltrexone and one nalmefene study. GABAergic/glutamatergic agents include baclofen and acamprosate. Sample drinking at baseline refers to studies enrolling participants who were not required to be abstinent prior to treatment initiation. Treatment ≤ 12 weeks denotes interventions delivered for 4–12 weeks. Conducted In North America includes studies from the United States and Canada. High risk of bias was categorised based on the Cochrane RoB 2, JBI, and conflict‐of‐interest/funding assessments. Low/medium risk of bias was categorised based on the Cochrane RoB 2, JBI, and conflict‐of‐interest/funding assessments. Sample comorbidity (*Y*) indicates studies where all participants had a comorbid condition (e.g., Major Depression or Cocaine Use Disorder). Sample comorbidity (*N*) indicates samples without systematic comorbidity, though individual participants may have had co‐occurring conditions. For subgroups defined by sample abstinent at baseline, treatment > 12 weeks, and not conducted in North America, too few studies compared treatment to control on consumption measures to permit subgroup analysis.

In outcomes with low/medium RoB, pooled estimates suggested small beneficial effects on overall efficacy (SMD = 0.18, *p* = 0.001, 95% CI [0.08, 0.29]) and consumption frequency (SMD = 0.21, *p* = 0.001, 95% CI [0.09, 0.33]). Sex‐moderator analyses were small and imprecise for overall efficacy (ΔSMD = 0.03, *p* = 0.79, 95% CI [−0.19, 0.25]), consumption frequency (ΔSMD = 0.11, *p* = 0.38, 95% CI [−0.14, 0.36]), and consumption quantity (ΔSMD = −0.19, *p* = 0.33, 95% CI [−0.60, 0.19]).

Compared to control, opioid receptor agonists (naltrexone and nalmefene) demonstrated small but positive treatment effects on overall efficacy (SMD = 0.17, *p* < 0.001, 95% CI [0.04, 0.30]), consumption frequency (SMD = 0.18, *p* = 0.001, 95% CI [0.05, 0.31]) and consumption quantity (SMD = 0.20, *p* = 0.02, 95% CI [0.03, 0.37]). Sex‐moderator analyses were small and imprecise, with confidence intervals including zero for overall efficacy (ΔSMD = 0.09, *p* = 0.24, 95% CI [−0.06, 0.24]), consumption frequency (ΔSMD = 0.15, *p* = 0.067, 95% CI [−0.01, 0.31]), and consumption quantity (ΔSMD = −0.31, *p* = 0.13, 95% CI [−0.70, 0.09]).

For studies conducted in North America, pooled estimates suggested small beneficial effects on overall efficacy (SMD = 0.12, *p* = 0.040, 95% CI [0.00, 0.24]) and consumption quantity (SMD = 0.17, *p* = 0.030, 95% CI [0.02, 0.32]). Sex‐moderator analyses were generally imprecise for both overall efficacy (ΔSMD = 0.12, *p* = 0.08, 95% CI [−0.01, 0.25]) and consumption quantity (ΔSMD = 0.01, *p* = 0.98, 95% CI [−0.36, 0.37]). For consumption frequency, the main effect was small and imprecise (SMD = 0.13, *p* = 0.058, 95% CI [−0.01, 0.26]), but the sex‐moderator model suggested an advantage in male participants (ΔSMD = 0.15, *p* = 0.049, 95% CI [0.01, 0.23]).

All remaining subgroup comparisons showed small or imprecise effects in both the main and sex‐moderated models, with confidence intervals including zero, indicating no clear evidence of subgroup‐specific differences.

#### 3.3.3 Sensitivity Analyses

When the single non‐randomised study was removed, the pooled effect comparing treatment to control remained similar in magnitude, though the CI included zero (SMD = 0.24, *p* = 0.076, 95% CI [−0.03, 0.51]; 11 trials; *I* ^2^ = 77%) and the sex‐moderator model remained small and imprecise (ΔSMD = 0.11, *p* = 0.33, 95% CI [−0.11, 0.34]).

For within‐subject change, sufficient data were available to conduct separate analyses for randomised and non‐randomised studies. Among RCTs, the pooled estimate indicated a significant reduction in consumption (SMCC = 1.44, *p* < 0.001, 95% CI [1.03, 1.85]; 11 trials; *I* ^2^ = 91%) but there was still no meaningful sex moderation (ΔSMCC = −0.14, *p* = 0.41, 95% CI [−0.49, 0.20]). Similar results were observed for non‐randomised studies, where the pooled effect also showed a significant reduction in consumption (SMCC = 1.20, *p* < 0.001, 95% CI [0.74, 1.65]; 4 studies; *I* ^2^ = 79%), with no evidence of sex moderation (ΔSMCC = −0.14, *p* = 0.38, 95% CI [−0.46, 0.18]).

#### 3.3.4 Regression Analyses

Meta‐regression analyses were conducted with each moderator first examined independently, followed by interaction models including sex × moderator terms (Table S4).

Across analyses, baseline severity, sample age, and treatment duration were associated with small effect sizes, and the 95% confidence intervals for all estimates included zero, indicating no clear evidence of meaningful moderation.

#### 3.3.5 Publication Bias

Publication bias is unlikely to have meaningfully influenced the observed treatment effects (see Figure S6 for full results, plots and GRADE assessments for the three primary outcomes). Power analyses based on the drug vs. control models for continuous consumption outcomes showed 299 participants (80% power, *α* = 0.05) per group would be required to detect the observed overall effect (SMD = 0.23); detecting the observed sex difference (ΔSMD = 0.05) would require ~6358 per group. The median observed power across studies was 13.7%.

### 3.4 Narrative Synthesis

#### 3.4.1 GABAergic and Glutamatergic Modulators

Garbutt et al. [54] found no treatment effect or sex interaction at 30 mg of *baclofen* over 12 weeks. In contrast, Garbutt et al. [55] found that baclofen doses (30 and 90 mg groups) reduced *Heavy Drinking Days* and increased *Abstinent Days* with significant sex interactions; female participants receiving 30 mg improved more than those on placebo; this was not observed in males. Morley et al. [60] also reported significant *Abstinent Days* increase in female participants at 30–75 mg doses. Reynaud et al. [64] found no overall treatment effect at 180 mg, though female participants receiving baclofen had significantly higher *Abstinence Rates* than those on placebo. Differences in sample severity and recruitment context may explain this heterogeneity. Garbutt et al. [54] recruited from the community and reported mild baseline severity, whereas Reynaud [64] drew from specialist hospitals. Additionally, adverse event profiles varied by sex. Garbutt et al. [55] found that female participants reported more sedation and fatigue, particularly at higher doses, and were more likely to discontinue treatment. However, no sex differences were found in baclofen concentrations, suggesting the increased sensitivity in female patients may reflect a pharmacodynamic effect.

Fertig et al. [52] evaluated *levetiracetam* in a 10‐week trial and found no significant reductions in *Heavy Drinking Days* and no sex‐by‐treatment interaction. Fatigue was the only adverse event more common in the active group; dose or sample characteristics were suggested as possible contributors to the null findings.

#### 3.4.2 Serotonergic and Dopaminergic Modulators

Two trials assessed *citalopram* (40 mg daily for 12 weeks). Naranjo et al. [61] recruited a mild/moderate AUD sample, while Adamson et al. [47] studied AUD patients with co‐occurring major depression. Neither trial found significant differences between citalopram and placebo on *Abstinent Days*, nor significant sex‐by‐treatment interactions. Naranjo et al. [61] found a sex‐specific effect for citalopram when controlling for baseline depression and anxiety. Male participants showed a significantly greater reduction in *Drinks per Day* than female participants. However, this finding is complicated by the lack of an effect on *Drinks per Drinking Day* (a measure that may better capture true drinking quantity by excluding abstinent days). Furthermore, female participants had fewer *Drinks per Day* at baseline; this limited scope for improvement may also contribute to the lack of a treatment effect seen in this group.

Wiesbeck et al. [66] conducted a 6‐month RCT comparing *flupenthixol* to placebo. Flupenthixol was associated with higher relapse rates overall, driven by significantly worse outcomes in male participants. Although female participants on flupenthixol reported fewer relapses than those on placebo, this difference was not statistically significant.

Two trials assessed *varenicline* (2 mg daily) in AUD in participants. O'Malley et al. [62] and Bold et al. [49] reported findings from the same 16‐week placebo‐controlled trial and found that varenicline reduced alcohol consumption, but differences from placebo were not statistically significant. A sex‐by‐treatment interaction on *Heavy Drinking Days* emerged at 16 weeks, but this interaction was not maintained at the one‐year follow‐up. Male participants reported significantly greater *No Heavy Drinking Days* compared to placebo at both end of treatment and one‐year follow‐up, which was not seen in female participants. Female participants were more likely to report adverse events and were less likely to adhere to the medication, which may have contributed to the lack of observed effects. Falk et al. [51] found that varenicline significantly reduced *Heavy Drinking Days*, *Drinks per Day* and *per Drinking Day* compared to placebo over 13 weeks, though no sex moderation was observed. That study identified several other potential moderators of treatment response, including age, years of drinking, smoking and treatment goals.

#### 3.4.3 Opioid Receptor Agonists

Hashimoto et al. [57] found that *nalmefene* (10–20 mg) significantly reduced *Heavy Drinking Days* compared to placebo. No sex‐by‐treatment interaction was observed. While the authors identified smoking, age and age of AUD onset as significant predictors of treatment response, sex did not moderate outcomes.

Garbutt et al. [53] reported significant reductions in *Heavy Drinking Days* in a 380 mg injectable *naltrexone* group only among male participants. Baros et al. [48] found that 100 mg oral naltrexone significantly increased *Abstinent Days* and reduced *Heavy Drinking Days* and *Drinks per Drinking Day* for male, but not female participants. Similarly, Pettinati et al. [63] reported a significant sex‐by‐treatment interaction for *Abstinent Rates* among individuals with co‐occurring CUD; male participants receiving naltrexone were significantly less likely to relapse than placebo, and female participants receiving naltrexone had greater relapse rates than placebo (non‐significant). Tolerability may have influenced outcomes. Garbutt et al. [53] noted that female participants experienced significantly more nausea, particularly at higher doses. Baros et al. [48] also reported greater nausea and sleep disturbances among female participants, and Pettinati et al. [63] observed lower adherence and higher dropout rates among female participants.

Three studies found no evidence of sex differences in naltrexone response. Yoon et al. [45], an 8‐week open‐label study using 50 mg oral naltrexone, observed significant reductions in *Drinks per Drinking Day* across the sample, with no interaction by sex. This study employed split dosing and antiemetic strategies, which may have improved tolerability and limited sex‐related differences. Greenfield et al. [68] examined 100 mg oral naltrexone and found no significant effects on *Abstinent Days* or moderation by sex. Goldstein [56] evaluated 380 mg injectable naltrexone in a homeless sample receiving specialist counselling and found positive treatment effects and no sex differences. It may be that specialist intervention attenuated possible sex differences. Limited reporting of side effects and adherence in these studies makes it unclear whether these factors were negligible or simply under‐evaluated.

## 4 Discussion

This systematic review and meta‐analysis aimed to evaluate whether the effectiveness of AUD pharmacotherapies varies by sex. Across 25,041 participants, female participants accounted for 25% of the total sample. Overall, pharmacotherapies were associated with modest benefits in reducing alcohol consumption and small effects for abstinence compared with control conditions. The estimated difference in treatment effect between male and female participants was close to zero, and the corresponding confidence intervals were narrow, indicating no clear evidence of a sex‐related difference in treatment response in the available data. Subgroup analyses and the narrative synthesis suggested possible sex differences for naltrexone, baclofen and studies conducted in North America. However, findings were based on limited and heterogeneous data.

Naltrexone was the most frequently studied pharmacotherapy. In the subgroup meta‐analysis of opioid receptor antagonists, moderate overall effects were observed for reducing both frequency and quantity of alcohol consumption, with no clear evidence that these effects differed between male and female participants. The narrative synthesis found that some trials reported greater efficacy in male participants, while others found no sex difference; studies reporting no difference included sample comorbidities, specialist interventions, or strategies to mitigate side effects. These factors are notable, as many studies reported poorer tolerability and higher attrition among female participants. Additionally, naltrexone's efficacy may be moderated by sex and gender related drinking motives. As a μ‐opioid receptor antagonist [70], naltrexone attenuates the positive reinforcing effects of alcohol [71, 72], making it particularly effective for individuals driven by reward‐seeking. This aligns with Mann et al. [73], who identified a ‘reward drinker’ phenotype that responded better to naltrexone than placebo. Some evidence suggests men are more likely to consume alcohol/substances for pleasure, reward and enhancement motives [74, 75, 76]. Studies in the narrative synthesis that found greater efficacy in male participants often included characteristics suggestive of reward sensitivity (e.g., stimulant use, smoking). These traits may reflect a subgroup that aligns more closely with naltrexone's mechanism. However, most studies in the current review did not assess drinking motives or craving profiles, limiting direct evaluation of this hypothesis.

Across several trials, female participants reported higher rates of adverse events, particularly nausea and fatigue, and in some cases showed lower adherence and greater dropout. These differences may be underpinned by sex‐specific pharmacokinetics and hormonal factors. Liu et al. [77] found that women exhibited higher metabolic ratios of 6β‐naltrexol to naltrexone than men, suggesting pharmacokinetic variability. Roche and King [22] further demonstrated that women, particularly during high‐oestrogen phases of the menstrual cycle, show heightened hormonal and subjective responses to naltrexone. Across studies that found no significant sex differences, adverse events and adherence data were rarely disaggregated by sex, limiting further analysis. Further sex‐stratified research evaluating naltrexone response while measuring hormones, tolerability and drinking motives is required.

The narrative synthesis revealed a pattern of greater baclofen efficacy in female participants, which may be related to sex and gender differences in drinking motives and psychiatric profiles. Typically, women are more likely to report drinking in response to stress or negative affect [78, 79], while men may be more driven by enhancement or social motives [80, 81]. Baclofen, a GABA‐B agonist with anxiolytic properties [82, 83], has been shown to be more effective in treating AUD in samples with comorbid anxiety [84]. This aligns with findings from Logge et al. [28], who observed baclofen disrupted the association between stress and drinking in women but not men, suggesting that its anxiolytic effects may be particularly relevant for emotionally motivated drinking. None of the baclofen trials included in the current review stratified drinking motives or baseline anxiety by sex, limiting exploration of this (or other) explanatory mechanisms.

Sex and gender are poorly defined and reported in alcohol treatment research, limiting subgroup analyses and intervention tailoring. A review of 86 youth alcohol studies found that none met the Sex and Gender Equity in Research (SAGER) guidelines [85, 86]. In this review, only 2 of 28 trials explained how they conceptualised or assessed sex. Of the 26 reports that used either term, 12 referred only to ‘sex’, 8 only to ‘gender’, and six used terms interchangeably. Studies in this review were selected based on availability of sex‐stratified data reporting, possibly introducing sampling bias, and terminology was assessed without the SAGER framework. However, the findings echo wider evidence that sex and gender are poorly conceptualised and reported in AUD research. There was no reporting of those who are non‐binary, intersex or trans, limiting what we know about their AUD treatment; reporting on these groups would be welcomed [87].

Trial design also limited the investigation of sex differences. Many trials apply exclusion criteria that disproportionately affect women (e.g., pregnancy, breastfeeding or lack of contraception) leading to smaller and more homogeneous female samples that do not represent the diversity of female experience and undermine the generalisability of findings [88]. While intended to minimise risk, these exclusions stem from concerns about reproductive risk, institutional liability, hormonal heterogeneity and assumptions about women's ability to reliably prevent pregnancy [89]. Even when women are included in trials, sex and/or gender are often not meaningfully considered in study design. For example, a common justification for not exploring sex differences in outcomes is the lack of observed differences in baseline characteristics. Yet, standard variables may not capture sex‐ or gender‐sensitive factors influencing treatment outcomes. For example, early substance use, depression, and involvement with child welfare were found to predict poorer outcomes among female participants in outpatient SUD treatment, whereas primary drug use frequency and age at intake were predictive among male participants [90]. Without intentional consideration of sex and gender throughout study design, trials risk overlooking meaningful subgroup differences and mischaracterising treatment effects.

### 4.1 Limitations

Only studies including both male and female participants were eligible. While this allowed for direct sex comparisons under consistent conditions, it excluded female‐ and male‐only trials that may offer valuable insights into pharmacotherapy response. It also excluded trans and gender diverse individuals, on whom we know there are limited studies, despite risk of harm [7]; where possible, studies should avoid reporting as ‘other’ to establish an evidence base [87]. Additionally, the review focused on clinical populations meeting diagnostic criteria for AUD or alcohol dependence. This may limit the applicability of findings to at‐risk populations, where women or other gender representations may be underdiagnosed or underrepresented in treatment settings. Meta‐analyses did not include time‐to‐event outcomes (e.g., time to lapse/relapse), which may be more sensitive to sustained treatment effects.

### 4.2 Recommendations

#### 4.2.1 Research

Future trials must be adequately powered to detect overall effects and sex‐based differences. Exclusion criteria related to reproductive status that disproportionately exclude women should be reconsidered. Trials should predefine sex and/or gender analyses; outcomes related to adherence, side effects, psychosocial variables, drinking motives and treatment goals should all be disaggregated by sex. Variables should include hormonal, reproductive and psychosocial factors; a broader range of baseline variables beyond age and education should be considered. Journals and funders should encourage the use of SAGER guidelines and promote open‐access repositories for sex‐disaggregated data. A consensus core outcome set would improve measurement standards and trial conduct [91, 92]. All prescribed AUD medications should be evaluated for sex‐specific efficacy. Large, well‐powered trials should evaluate sex differences in naltrexone and baclofen; naltrexone trials should assess dosing, tolerability, adherence and moderators such as drinking motives and hormonal influences; baclofen trials should consider stress‐ or negative affect–related drinking, baseline anxiety and psychiatric comorbidities.

#### 4.2.2 Practice

Medications like disulfiram, gabapentin, topiramate and antipsychotics lack evidence for women and should not be first‐line treatments. Naltrexone may be more effective in men; for women, consider side effects, anti‐emetics and split dosing. Baclofen may suit women, particularly with stress‐related drinking, but requires careful monitoring. All treatment plans should consider drinking motives, comorbidities and goals.

## 5 Conclusions

This review found no consistent evidence that treatment efficacy differs by sex. However, subgroup analyses and narrative findings suggest possible sex‐specific responses in certain contexts, particularly for naltrexone and baclofen. These patterns were more apparent in studies with adequate sex‐stratified analyses. Current evidence is insufficient to support broad conclusions about sex differences in pharmacotherapy response. Nonetheless, the findings highlight the need for adequately powered trials that account for sex‐ and gender‐considered variables. Improving the evidence base requires inclusive research practices, consistent outcome definitions and transparent reporting.

## Author Contributions

Each author certifies that their contribution to this work meets the standards of the International Committee of Medical Journal Editors.

## Funding

The authors have nothing to report.

## Conflicts of Interest

The authors declare no conflicts of interest.

## Supporting information


**Figure S1:** RoB 2 result.

**Figure S2:** Meta‐analysis of alcohol consumption change (between‐subjects, intervention vs. control).

**Figure S3:** Meta‐analysis of binary outcomes (abstinence/no HDD).

**Figure S4:** Meta‐analysis of within‐subject data.

**Figure S5:** Visualisation of findings for narrative synthesis.

**Figure S6:** Publication bias results.

**Data S1:** Supporting Information—Meta‐analysis R code.

**Table S1:** Search strategy terms.

**Table S2:** Summary of data conversions applied to extracted estimates.

**Table S3:** PRISMA checklist.

**Table S4:** Moderator effects and sex × moderator interactions by multi‐level meta‐regression analyses.

**Table S5:** Full text exclusions.

## Data Availability

The data that support the findings of this study are available from the corresponding author upon reasonable request.
