# Supplementary figures and images for: Effectiveness of Alcohol Use Disorder Pharmacotherapies by Sex: Systematic Review and Meta‐Analysis
Source: Drug Alcohol Rev. 2026 Jun 23;45(5):e70196. doi: 10.1111/dar.70196 (PMC13290497; doi:10.1111/dar.70196)

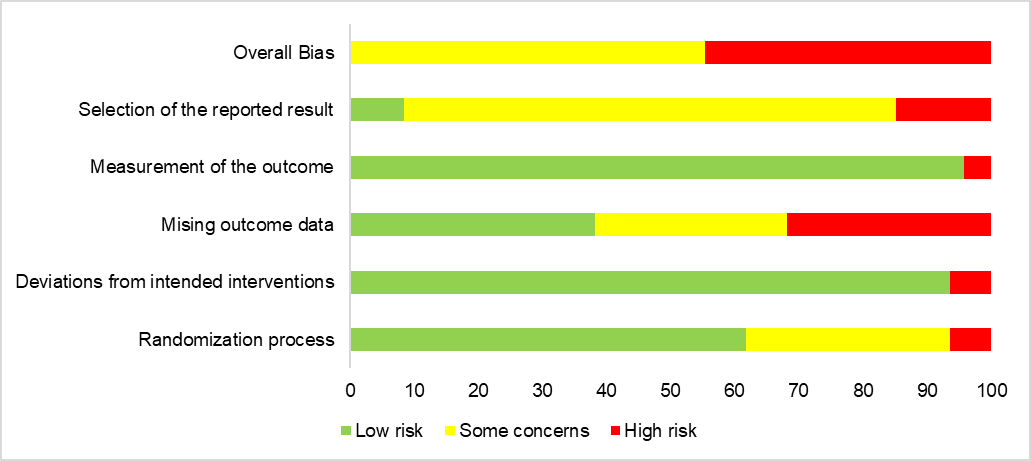
**Figure S1. RoB 2 Result**

Supplement: Supplementary file 1 — Figure S1: RoB 2 result. [file DAR-45-0-s010.docx]

Figure S3. Meta-Analysis of Binary Outcomes (Abstinence/No HDD)


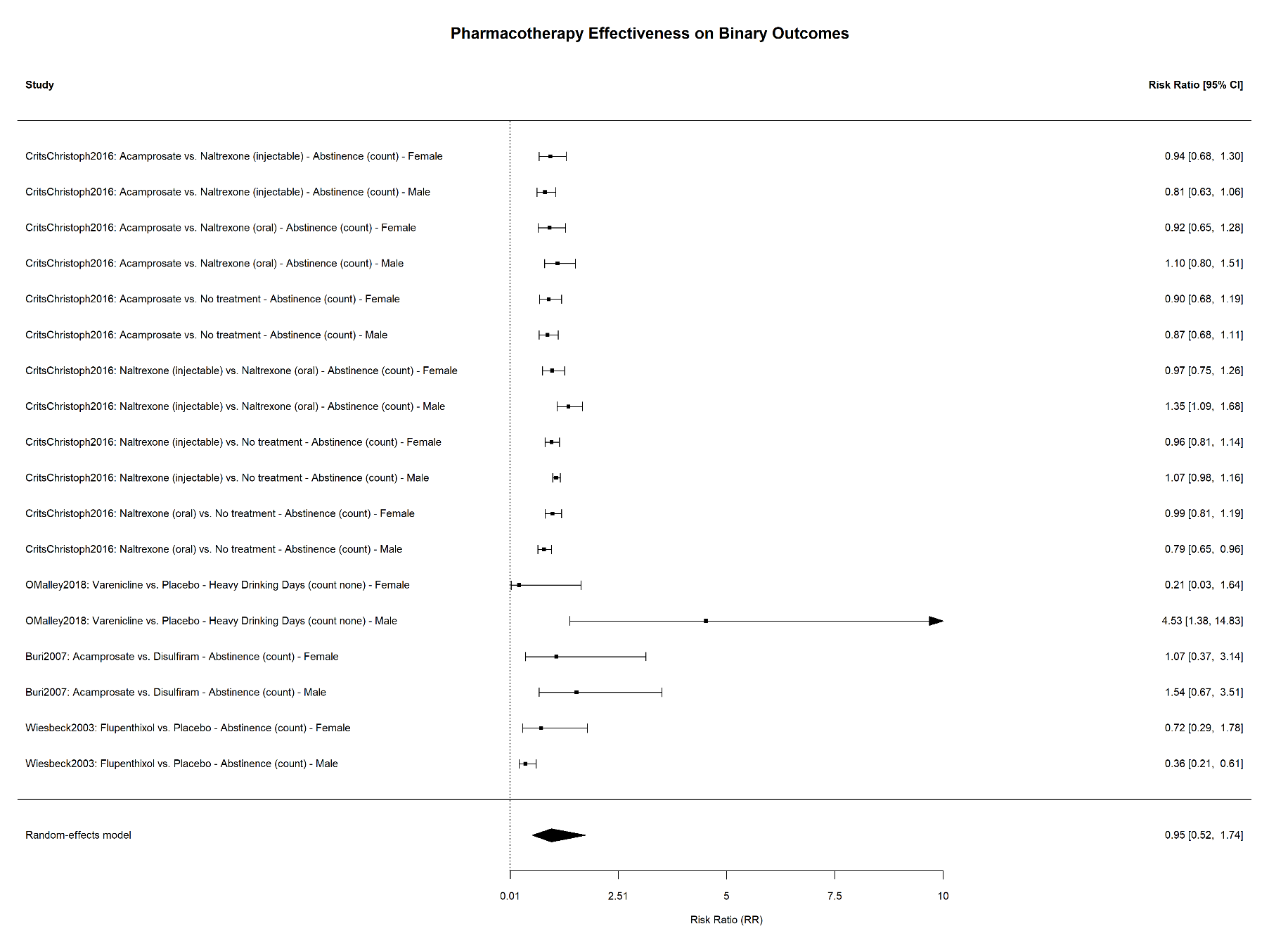

Supplement: Supplementary file 3 — Figure S3: Meta‐analysis of binary outcomes (abstinence/no HDD). [file DAR-45-0-s006.docx]

Figure S4. Meta-Analysis of Within-Subject Data


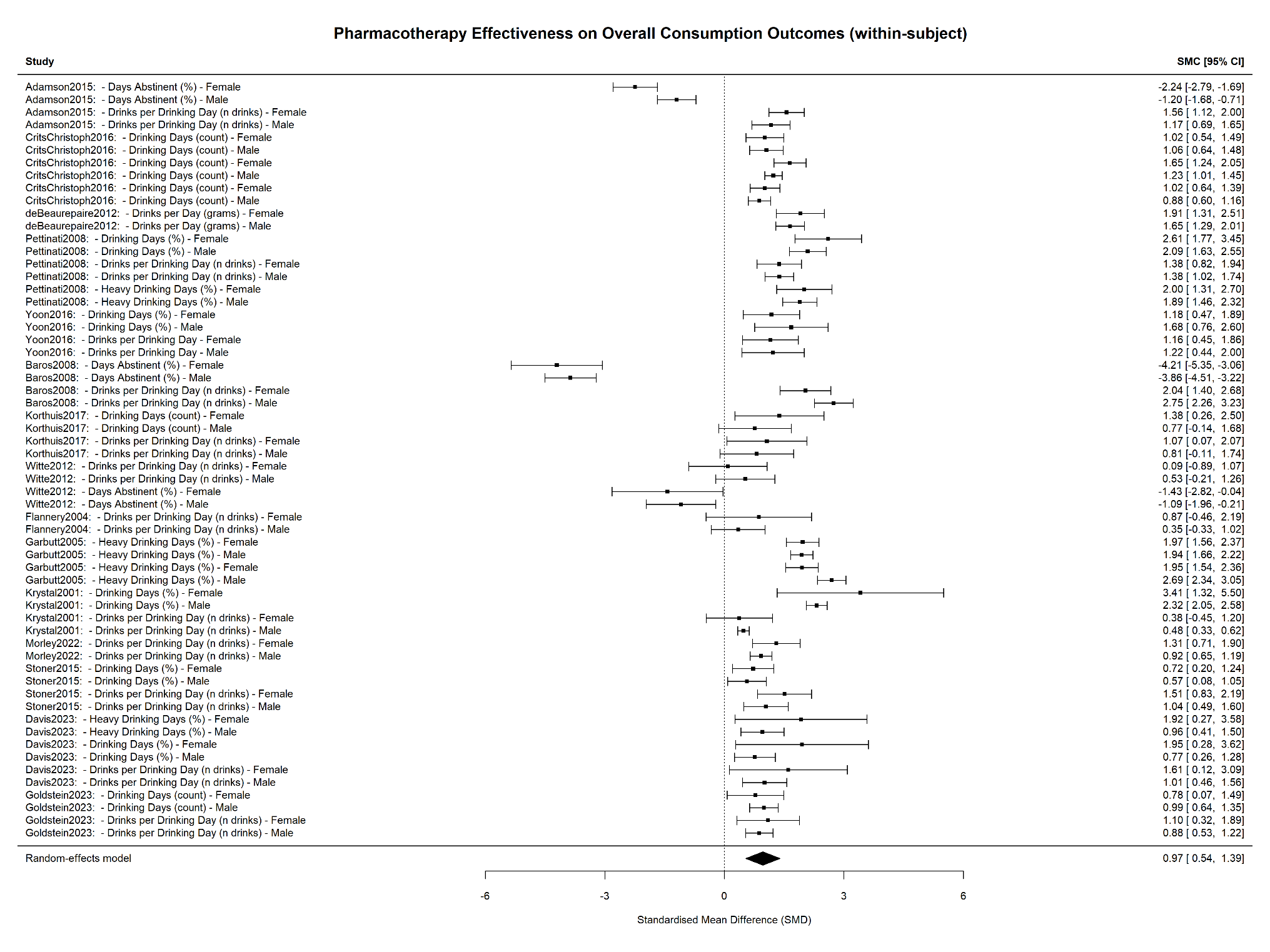

Supplement: Supplementary file 4 — Figure S4: Meta‐analysis of within‐subject data. [file DAR-45-0-s011.docx]

**
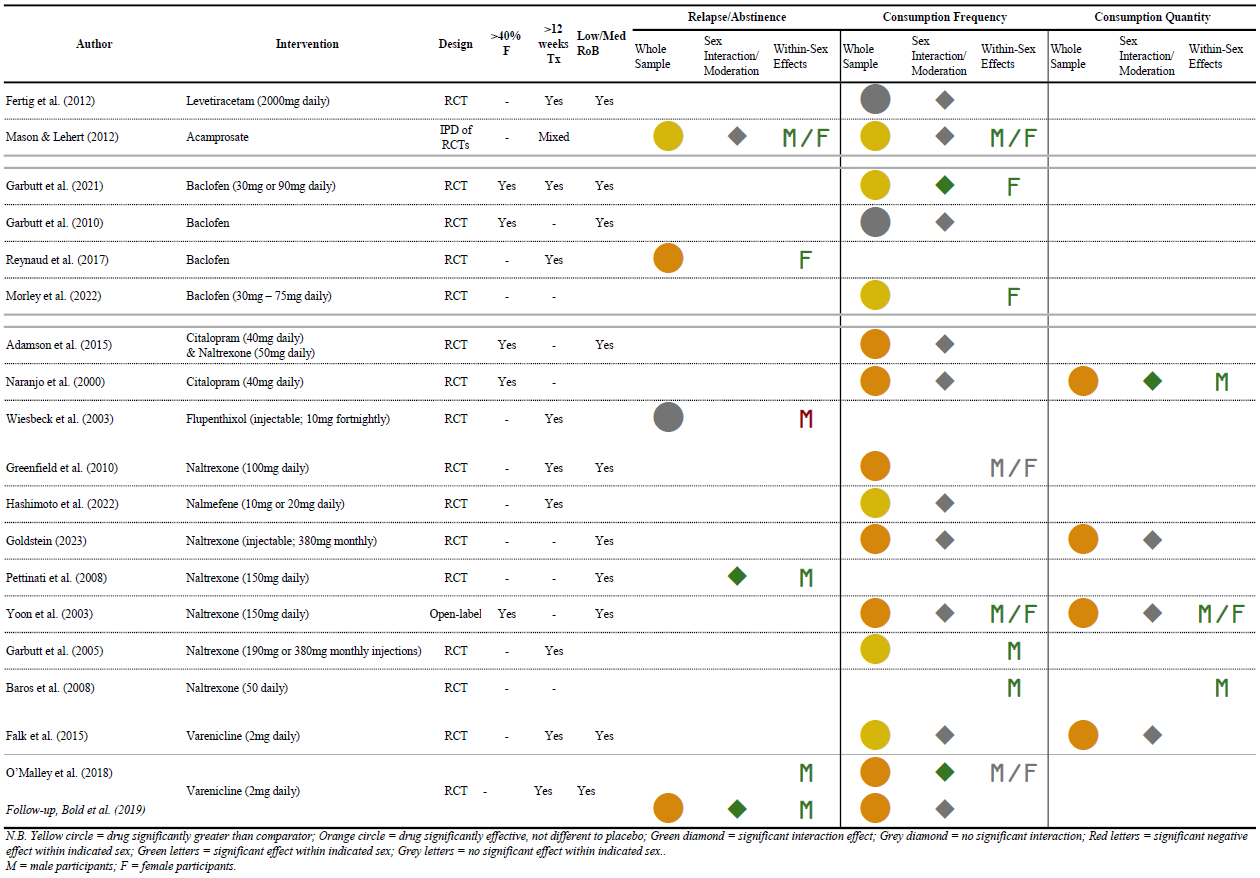
Figure S5. Visualisation of findings for narrative synthesis**

Supplement: Supplementary file 5 — Figure S5: Visualisation of findings for narrative synthesis. [file DAR-45-0-s009.docx]
