# Supplementary material for: Effectiveness of Alcohol Use Disorder Pharmacotherapies by Sex: Systematic Review and Meta‐Analysis
Source: Drug Alcohol Rev. 2026 Jun 23;45(5):e70196. doi: 10.1111/dar.70196 (PMC13290497; doi:10.1111/dar.70196)

Figure S2. Meta-Analysis of Alcohol Consumption Change (Between-Subjects, Intervention vs. Control)


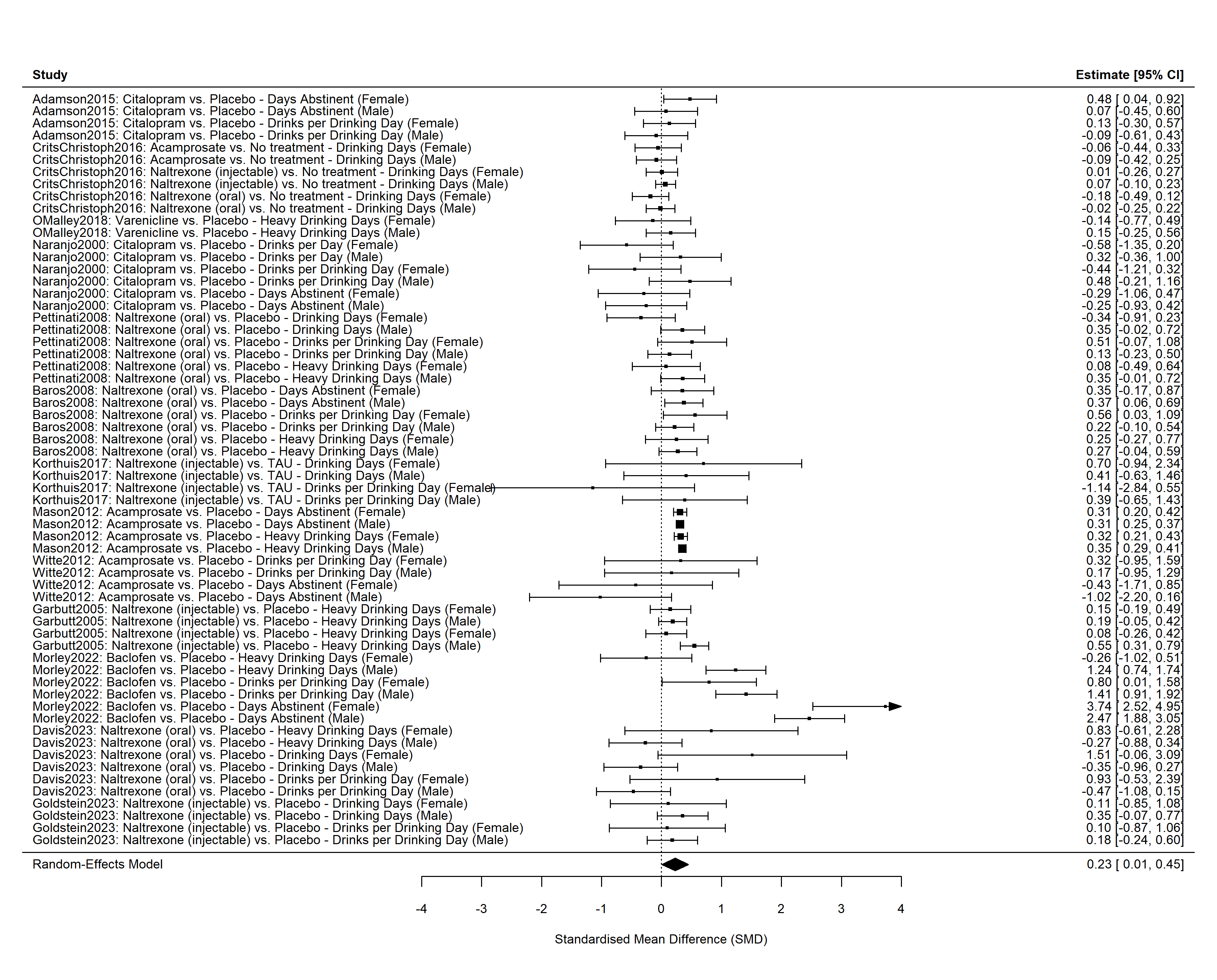

Supplement: Supplementary file 2 — Figure S2: Meta‐analysis of alcohol consumption change (between‐subjects, intervention vs. control). [file DAR-45-0-s001.docx]
