# Supplementary material for: Effectiveness of Alcohol Use Disorder Pharmacotherapies by Sex: Systematic Review and Meta‐Analysis
Source: Drug Alcohol Rev. 2026 Jun 23;45(5):e70196. doi: 10.1111/dar.70196 (PMC13290497; doi:10.1111/dar.70196)
Supplement: Supplementary file 6 — Figure S6: Publication bias results. [file DAR-45-0-s002.docx]

**Publication Bias Results**

Publication bias was assessed using funnel plots and Egger’s regression tests, with analyses aligned to the structure of the meta-analytic models.

*Between-Subject Continuous Outcomes (Treatment vs Control)*

In the MLM-based funnel plots, no clear evidence of asymmetry was observed (Figure S5). For both female and male participants, data points were relatively symmetrically distributed around the pooled effect size. There was some scatter among smaller studies, particularly in the female subgroup, but no consistent directional pattern indicative of publication bias. These visual patterns were consistent with Egger’s regression results from the SLM models, which indicated no significant asymmetry in the overall sample (z= -0.68, *p*=.49), female subgroup (z= 0.07, *p*=.94), or male subgroup (z= -0.67, *p*=.50). Neither visual inspection of the funnel plots nor the results of Egger’s tests provide evidence of systematic publication bias in the current meta-analyses; publication bias is unlikely to have meaningfully influenced the observed treatment effects.


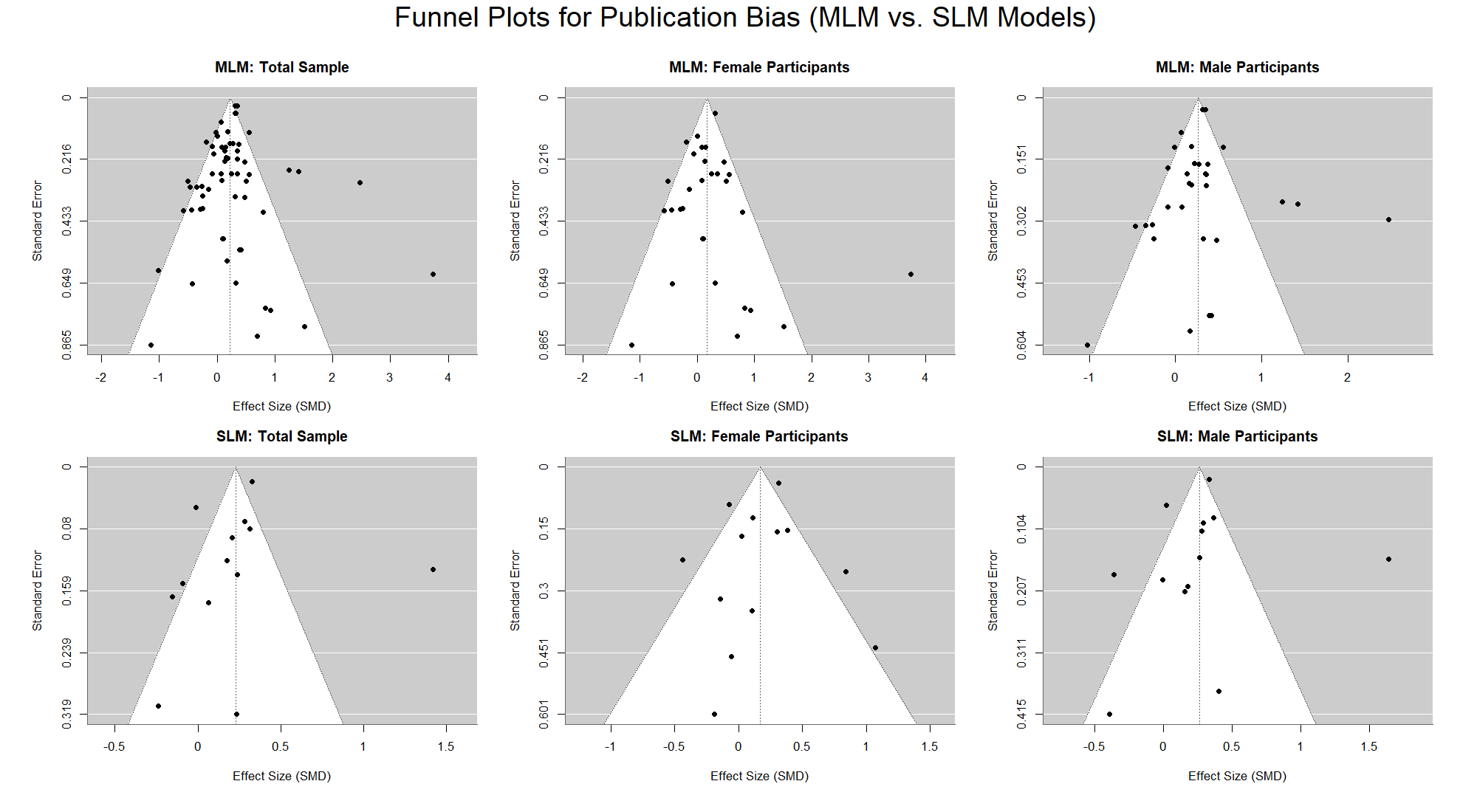


*Binary Outcomes*

Visual inspection of the funnel plot did not indicate clear asymmetry. Egger’s regression test was not statistically significant (z= 0.74, p=.46), suggesting no statistical evidence of small-study effects. However, interpretation should be cautious given the small number of studies and substantial heterogeneity observed in these models.
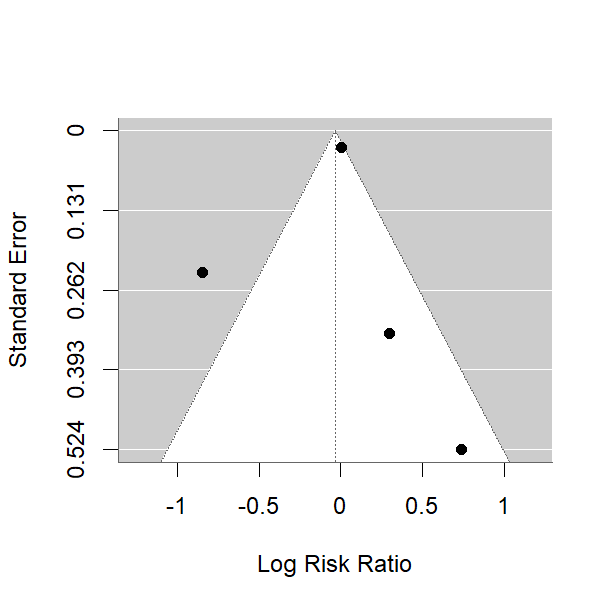


*Within-Subject Continuous Outcomes*

Visual inspection of the funnel plot suggested some asymmetry, with smaller studies tending to report larger positive effects. Egger’s regression test was not statistically significant (z= −1.65, p=.10). Although this does not provide statistical evidence of publication bias, the possibility of small-study effects cannot be excluded, particularly in the context of high between-study heterogeneity.


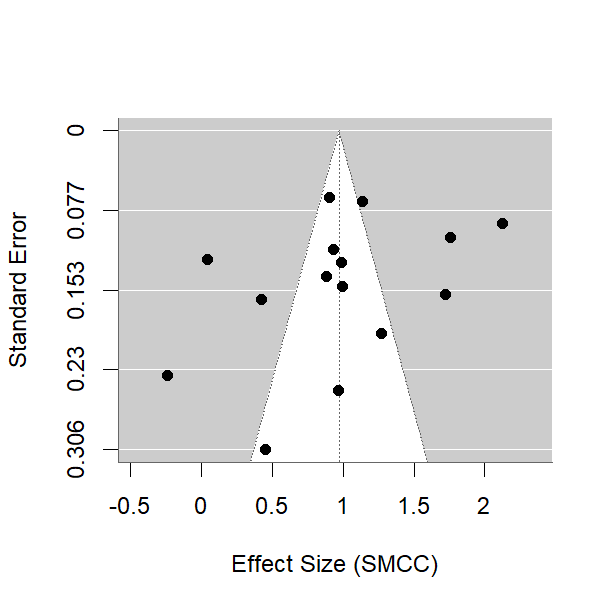


**Grade assessment results**

For continuous between-group alcohol consumption outcomes (treatment vs control), the certainty of evidence was rated as low. Downgrading was applied due to serious concerns regarding risk of bias (the majority of outcomes derived from high-risk studies), substantial heterogeneity (I² = 88.84%), and serious imprecision, as the confidence interval narrowly excluded the null (SMD 0.23, 95% CI 0.01–0.45). The evidence was otherwise direct, and publication bias was not detected.

For binary outcomes, the certainty of evidence was rated as very low. This rating reflected very serious concerns regarding risk of bias, as all contributing studies were judged to be at high risk of bias, as well as very serious imprecision due to wide confidence intervals encompassing both meaningful benefit and harm (RR 0.96, 95% CI 0.52–1.74). Substantial statistical heterogeneity (I² = 94.79%) further reduced confidence in the pooled estimate. The evidence was considered direct, and no evidence of publication bias was detected; however, these factors did not offset the overall low certainty.

For within-subject continuous outcomes, the certainty of evidence was rated as moderate. Although a proportion of contributing studies were at high risk of bias and heterogeneity was considerable (I² = 96.62%), the pooled effect estimate was relatively precise and consistently favoured treatment (SMCC 0.97, 95% CI 0.54–1.39). The evidence was direct. Visual inspection of the funnel plot suggested some asymmetry, with smaller studies tending to report larger positive effects. Confidence was therefore downgraded for risk of bias and inconsistency, but not for imprecision or indirectness; potential publication bias was considered but not judged sufficient to warrant further downgrading.
