# Supplementary material for: Effectiveness of Alcohol Use Disorder Pharmacotherapies by Sex: Systematic Review and Meta‐Analysis
Source: Drug Alcohol Rev. 2026 Jun 23;45(5):e70196. doi: 10.1111/dar.70196 (PMC13290497; doi:10.1111/dar.70196)
Supplement: Supplementary file 7 — Data S1: Supporting Information—Meta‐analysis R code. [file DAR-45-0-s003.docx]

**Supplementary Materials – Meta-Analysis R Code**

library(readxl)

library(metafor)

library(dmetar)

library(janitor)

library(dplyr)

####load and clean data

updated_meta_sheet <- read_excel("C:/Users/Juliette/OneDrive - Liverpool John Moores University/Documents/Systematic Review/07 Analysis/march final met/meta sheet final.xlsx")

data_file<-clean_names(updated_meta_sheet)

###keep only continuous variables

data_cont<-subset(data_file, data_file$summary_statistic=="Standardised Mean Difference")

##ensure all continuous variables store data as numeric (debug excel error of non-numeric values)

data_cont$mean1<-as.numeric(data_cont$mean1)

data_cont$mean2<-as.numeric(data_cont$mean2)

data_cont$sd1<-as.numeric(data_cont$sd1)

data_cont$sd2<-as.numeric(data_cont$sd2)

#########filter data to BS effects

BSall<-subset(data_cont, data_cont$method=="between")

###calculate BS SMD/effect size

BSall<-escalc(measure = "SMD",

m1i = mean1,

m2i = mean2,

sd1i = sd1,

sd2i = sd2,

n1i = n1,

n2i = n2,

data = BSall)

####add unique identifier for each individual effect

data_cont$uid<-1:nrow(data_cont)

BSall$uid<-1:nrow(BSall)

#calculate unweighted mean effect size across studies

mean(BSall$yi)

##reverse variables with "y" to ensure aligned direction for positive treatment effect

BSall$yi_final<-ifelse(BSall$reverse=="y", -1*BSall$yi, BSall$yi)

##unweighted mean SMD after reversing

mean(BSall$yi_final)

##check group_2 comparators to identify drug vs drug and drug vs placebo(or other) groups

table(BSall$group_2)

###split drug vs. placebo and drug vs. drug

BSall$vs_PLA<-ifelse(BSall$group_2%in%c("Notreatment",

"Placebo",

"PlaceboEscitalopram",

"PlaceboNaltrexone",

"TAU"), "yes", "no")

BSvsPLA<-subset(BSall, BSall$vs_PLA=="yes")

##################################### BSall meta

BSall_freq <- subset(BSall, outcome_domain == "Frequency")

BSall_quant <- subset(BSall, outcome_domain == "Quantity")

length(unique(BSall$id))

length(unique(BSall_freq$id))

length(unique(BSall_quant$id))

length(unique(BSall$uid))

length(unique(BSall_freq$uid))

length(unique(BSall_quant$uid))

meta_BSall_OE_main<-rma.mv(yi = yi_final,

V = vi,

random = ~ 1 | id/uid,

data = BSall)

meta_BSall_freq_main<-rma.mv(yi = yi_final,

V = vi,

random = ~ 1 | id/uid,

data = BSall_freq)

meta_BSall_quant_main<-rma.mv(yi = yi_final,

V = vi,

random = ~ 1 | id/uid,

data = BSall_quant)

meta_BSall_OE_sex<-rma.mv(yi = yi_final,

V = vi,

random = ~ 1 | id/uid,

data = BSall,

mods = ~sex)

meta_BSall_freq_sex<-rma.mv(yi = yi_final,

V = vi,

random = ~ 1 | id/uid,

data = BSall_freq,

mods = ~sex)

meta_BSall_quant_sex<-rma.mv(yi = yi_final,

V = vi,

random = ~ 1 | id/uid,

data = BSall_quant,

mods = ~sex)

summary(meta_BSall_OE_main)

summary(meta_BSall_freq_main)

summary(meta_BSall_quant_main)

summary(meta_BSall_OE_sex)

summary(meta_BSall_freq_sex)

summary(meta_BSall_quant_sex)

mlm.variance.distribution(meta_BSall_OE_main)

mlm.variance.distribution(meta_BSall_freq_main)

mlm.variance.distribution(meta_BSall_quant_main)

meta_BSvsPLA_OE_main<-rma.mv(yi = yi_final,

V = vi,

random = ~ 1 | id/uid,

data = BSvsPLA,

)

meta_BSvsPLA_OE_sex<-rma.mv(yi = yi_final,

V = vi,

random = ~ 1 | id/uid,

data = BSvsPLA,

mods = ~sex

)

###################### BSvsPLA meta

meta_BSvsPLA_OE_main<-rma.mv(yi = yi_final,

V = vi,

random = ~ 1 | id/uid,

data = BSvsPLA,

)

##################################################################### POWER CALC

install.packages('pwr')

library(pwr)

?pwr.t.test

#######################power overall

effect_pooled <- meta_BSvsPLA_OE_main$b# pooled effect size dragged from the model

alpha <- 0.05

vi <- BSvsPLA$vi #### variance of samples

# Compute standard errors

sei <- sqrt(vi)

# Compute power for each study

z <- qnorm(1 - alpha / 2)

power <- 1 - pnorm(z - effect_pooled / sei) + pnorm(-z - effect_pooled / sei)

BSvsPLA$power <- power

median(BSvsPLA$power)

############minimum needed

power_needed<-pwr.t.test(d = meta_BSvsPLA_OE_main$b, sig = .05, power = .80, type = "two.sample")

power_needed

#####################################################################################################

meta_BSvsPLA_OE_sex<-rma.mv(yi = yi_final,

V = vi,

random = ~ 1 | id/uid,

data = BSvsPLA,

mods = ~sex

)

#######################power sex

effect_pooled <- meta_BSvsPLA_OE_sex$b# pooled effect size dragged from the model

# Extract intercept and sex effect

b_intercept <- as.numeric(meta_BSvsPLA_OE_sex$b[1])

b_sex <- as.numeric(meta_BSvsPLA_OE_sex$b[2])

# convert to numeric: 0 = Male, 1 = Female

BSvsPLA$sex_numeric <- ifelse(BSvsPLA$sex == "Male", 1, 0)

alpha <- 0.05

z <- qnorm(1 - alpha / 2)

sei <- sqrt(BSvsPLA$vi)

BSvsPLA$sex_specific_effect <- b_intercept + b_sex * BSvsPLA$sex_numeric

BSvsPLA$power_sex_specific <- 1 - pnorm(z - BSvsPLA$sex_specific_effect / sei) +

pnorm(-z - BSvsPLA$sex_specific_effect / sei)

summary(BSvsPLA$power_sex_specific)

median(BSvsPLA$power)

BSvsPLA$sex_specific_effect <- b_intercept + b_sex * BSvsPLA$sex_numeric

alpha <- 0.05

vi <- BSvsPLA$vi

sei <- sqrt(vi)

z <- qnorm(1 - alpha / 2)

power <- 1 - pnorm(z - effect_pooled / sei) + pnorm(-z - effect_pooled / sei)

BSvsPLA$power <- power

median(BSvsPLA$power)

############minimum needed

power_needed<-pwr.t.test(d = meta_BSvsPLA_OE_sex$b, sig = .05, power = .80, type = "two.sample")

power_needed

####################################################################################

b_sex <- as.numeric(meta_BSvsPLA_OE_sex$b[2])# the estimated difference between males and females

power_sex_difference <- pwr.t.test(

d = b_sex,# effect size: difference in SMDs between sexes

sig.level = 0.05, # standard alpha

power = 0.80, # desired power

type = "two.sample",# comparing two groups (male vs female)

alternative = "two.sided"

)

power_sex_difference

#####################################################################################################

meta_BSvsPLA_freq_main<-rma.mv(yi = yi_final,

V = vi,

random = ~ 1 | id/uid,

data = BSvsPLA,

subset = outcome_domain =="Frequency"

)

meta_BSvsPLA_freq_sex<-rma.mv(yi = yi_final,

V = vi,

random = ~ 1 | id/uid,

data = BSvsPLA,

subset = outcome_domain =="Frequency",

mods = ~sex

)

meta_BSvsPLA_quant_main<-rma.mv(yi = yi_final,

V = vi,

random = ~ 1 | id/uid,

data = BSvsPLA,

subset = outcome_domain =="Quantity"

)

meta_BSvsPLA_quant_sex<-rma.mv(yi = yi_final,

V = vi,

random = ~ 1 | id/uid,

data = BSvsPLA,

subset = outcome_domain =="Quantity",

mods = ~sex

)

summary(meta_BSvsPLA_OE_main)

summary(meta_BSvsPLA_OE_sex)

summary(meta_BSvsPLA_freq_main)

summary(meta_BSvsPLA_freq_sex)

summary(meta_BSvsPLA_quant_main)

summary(meta_BSvsPLA_quant_sex)

###### I2 & n id/uid

mlm.variance.distribution(meta_BSvsPLA_OE_main)

mlm.variance.distribution(meta_BSvsPLA_freq_main)

mlm.variance.distribution(meta_BSvsPLA_quant_main)

BSvsPLA_freq <- subset(BSall, outcome_domain == "Frequency")

BSvsPLA_quant <- subset(BSall, outcome_domain == "Quantity")

length(unique(BSvsPLA$uid))

length(unique(BSvsPLA_freq$uid))

length(unique(BSvsPLA_quant$uid))

length(unique(BSvsPLA$id))

length(unique(BSvsPLA_freq$id))

length(unique(BSvsPLA_quant$id))

###################################### WITHIN SUBJECT META

##split data into within effects

within<-subset(data_cont, data_cont$method=="within")

str(data_cont)

within<-escalc(measure = "SMCC",

m1i = mean2,

m2i = mean1,

sd1i = sd2,

sd2i = sd1,

ni = n1,

ri = cor,

data = within)

data_cont$uid<-1:nrow(data_cont)

within$uid<-1:nrow(within)

mean(within$yi)

within$yi_final1<-ifelse(within$reverse=="y", -1*within$yi, within$yi)

mean(within$yi_final1)

within$yi_final <- -1*within$yi_final1

mean(within$yi_final)

length(unique(within$id))

length(unique(within$uid))

within_freq <-subset(within, outcome_domain == "Frequency")

within_quant <-subset(within, outcome_domain == "Quantity")

length(unique(within_freq$id))

length(unique(within_quant$id))

length(unique(within_freq$uid))

length(unique(within_quant$uid))

meta_WS_OE_main<-rma.mv(yi = yi,

V = vi,

random = ~ 1 | id/uid,

data = within,

)

meta_WS_freq_main<-rma.mv(yi = yi,

V = vi,

random = ~ 1 | id/uid,

data = within_freq

)

meta_WS_quant_main<-rma.mv(yi = yi,

V = vi,

random = ~ 1 | id/uid,

data = within_quant

)

meta_WS_OE_sex<-rma.mv(yi = yi,

V = vi,

random = ~ 1 | id/uid,

data = within,

mods = ~sex

)

meta_WS_freq_sex<-rma.mv(yi = yi,

V = vi,

random = ~ 1 | id/uid,

data = within_freq,

mods = ~sex

)

meta_WS_quant_sex<-rma.mv(yi = yi,

V = vi,

random = ~ 1 | id/uid,

data = within_quant,

mods = ~sex

)

summary(meta_WS_OE_main)

summary(meta_WS_freq_main)

summary(meta_WS_quant_main)

summary(meta_WS_OE_sex)

summary(meta_WS_freq_sex)

summary(meta_WS_quant_sex)

mlm.variance.distribution(meta_WS_OE_main)

mlm.variance.distribution(meta_WS_freq_main)

mlm.variance.distribution(meta_WS_quant_main)

############## BSvsPLA Subgroup Analyses

#split data into subgroups & check id/uid n (id>3 for inclusion)

BSvsPLA_opioid <- subset(BSvsPLA, big_experimental_group == "Opioid")

length(unique(BSvsPLA_opioid$id))

length(unique(BSvsPLA_opioid$uid))

BSvsPLA_GABA <-subset(BSvsPLA, big_experimental_group == "GABAergicGlutamatergic")

length(unique(BSvsPLA_GABA$id))

length(unique(BSvsPLA_GABA$uid))

BSvsPLA_bAbstinent <- subset(BSvsPLA, drinking_at_baseline == "No")

length(unique(BSvsPLA_bAbstinent$id)) ## id = 3, cannot be included

length(unique(BSvsPLA_bAbstinent$uid))

BSvsPLA_bDrinking <- subset(BSvsPLA, drinking_at_baseline == "Yes")

length(unique(BSvsPLA_bDrinking$id))

length(unique(BSvsPLA_bDrinking$uid))

BSvsPLA$tx_length_cat <- ifelse(BSvsPLA$tx_length_weeks > 12, "Over_12_weeks", "Up_to_12_weeks")

BSvsPLA_short <- subset(BSvsPLA, tx_length_cat == "Up_to_12_weeks")

length(unique(BSvsPLA_short$id))

length(unique(BSvsPLA_short$uid))

BSvsPLA_long <- subset(BSvsPLA, tx_length_cat == "Over_12_weeks")

length(unique(BSvsPLA_long$id)) ## id = 3, cannot be included

length(unique(BSvsPLA_long$uid))

BSvsPLA$Northamerica<-ifelse(BSvsPLA$country1%in%c("USA",

"Canada",

"USA & Canada"), "yes", "no")

BSvsPLA_NorthAmerica <-subset(BSvsPLA, BSvsPLA$Northamerica =="yes")

length(unique(BSvsPLA_NorthAmerica$id))

length(unique(BSvsPLA_NorthAmerica$uid))

BSvsPLA_EuropeOther<-subset(BSvsPLA, BSvsPLA$Northamerica =="no")

length(unique(BSvsPLA_EuropeOther$id)) ## id = 3, cannot be included

length(unique(BSvsPLA_EuropeOther$uid))

BSvsPLA_HighROB <-subset(BSvsPLA, rob == "High")

length(unique(BSvsPLA_HighROB$id))

length(unique(BSvsPLA_HighROB$uid))

BSvsPLA_LowMedRob <-subset(BSvsPLA, rob == "Low/Medium")

length(unique(BSvsPLA_LowMedRob$id))

length(unique(BSvsPLA_LowMedRob$uid))

BSvsPLA_YComorbid<-subset(BSvsPLA, sample_comorbidity_presence1_65=="Yes")

length(unique(BSvsPLA_YComorbid$id))

length(unique(BSvsPLA_YComorbid$uid))

BSvsPLA_NComorbid<-subset(BSvsPLA, sample_comorbidity_presence1_65=="No")

length(unique(BSvsPLA_NComorbid$id))

length(unique(BSvsPLA_NComorbid$uid))

### naltrexone subgroup

meta_BSvsPLA_overallefficacy_main_opioid<-rma.mv(yi = yi_final,

V = vi,

random = ~ 1 | id/uid,

data = BSvsPLA_opioid,

)

meta_BSvsPLA_overallefficacy_sexmod_opioid<-rma.mv(yi = yi_final,

V = vi,

random = ~ 1 | id/uid,

data = BSvsPLA_opioid,

mods = ~sex)

meta_BSvsPLA_frequency_main_opioid<-rma.mv(yi = yi_final,

V = vi,

random = ~ 1 | id/uid,

data = BSvsPLA_opioid,

subset = outcome_domain =="Frequency"

)

meta_BSvsPLA_frequency_sexmod_opioid<-rma.mv(yi = yi_final,

V = vi,

random = ~ 1 | id/uid,

data = BSvsPLA_opioid,

subset = outcome_domain =="Frequency",

mods = ~sex)

meta_BSvsPLA_quantity_main_opioid<-rma.mv(yi = yi_final,

V = vi,

random = ~ 1 | id/uid,

data = BSvsPLA_opioid,

subset = outcome_domain =="Quantity"

)

meta_BSvsPLA_quantity_sexmod_opioid<-rma.mv(yi = yi_final,

V = vi,

random = ~ 1 | id/uid,

data = BSvsPLA_opioid,

subset = outcome_domain =="Quantity",

mods = ~sex)

### gaba subgroup

meta_BSvsPLA_overallefficacy_main_GABA<-rma.mv(yi = yi_final,

V = vi,

random = ~ 1 | id/uid,

data = BSvsPLA_GABA,

)

meta_BSvsPLA_overallefficacy_sexmod_GABA<-rma.mv(yi = yi_final,

V = vi,

random = ~ 1 | id/uid,

data = BSvsPLA_GABA,

mods = ~sex)

meta_BSvsPLA_frequency_main_GABA<-rma.mv(yi = yi_final,

V = vi,

random = ~ 1 | id/uid,

data = BSvsPLA_GABA,

subset = outcome_domain =="Frequency"

)

meta_BSvsPLA_frequency_sexmod_GABA<-rma.mv(yi = yi_final,

V = vi,

random = ~ 1 | id/uid,

data = BSvsPLA_GABA,

subset = outcome_domain =="Frequency",

mods = ~sex)

meta_BSvsPLA_quantity_main_GABA<-rma.mv(yi = yi_final,

V = vi,

random = ~ 1 | id/uid,

data = BSvsPLA_GABA,

subset = outcome_domain =="Quantity"

)

meta_BSvsPLA_quantity_sexmod_GABA<-rma.mv(yi = yi_final,

V = vi,

random = ~ 1 | id/uid,

data = BSvsPLA_GABA,

subset = outcome_domain =="Quantity",

mods = ~sex)

### bDrinking subgroup

meta_BSvsPLA_overallefficacy_main_bDrinking<-rma.mv(yi = yi_final,

V = vi,

random = ~ 1 | id/uid,

data = BSvsPLA_bDrinking,

)

meta_BSvsPLA_overallefficacy_sexmod_bDrinking<-rma.mv(yi = yi_final,

V = vi,

random = ~ 1 | id/uid,

data = BSvsPLA_bDrinking,

mods = ~sex)

meta_BSvsPLA_frequency_main_bDrinking<-rma.mv(yi = yi_final,

V = vi,

random = ~ 1 | id/uid,

data = BSvsPLA_bDrinking,

subset = outcome_domain =="Frequency"

)

meta_BSvsPLA_frequency_sexmod_bDrinking<-rma.mv(yi = yi_final,

V = vi,

random = ~ 1 | id/uid,

data = BSvsPLA_bDrinking,

subset = outcome_domain =="Frequency",

mods = ~sex)

meta_BSvsPLA_quantity_main_bDrinking<-rma.mv(yi = yi_final,

V = vi,

random = ~ 1 | id/uid,

data = BSvsPLA_bDrinking,

subset = outcome_domain =="Quantity"

)

meta_BSvsPLA_quantity_sexmod_bDrinking<-rma.mv(yi = yi_final,

V = vi,

random = ~ 1 | id/uid,

data = BSvsPLA_bDrinking,

subset = outcome_domain =="Quantity",

mods = ~sex)

### short subgroup

meta_BSvsPLA_overallefficacy_main_short<-rma.mv(yi = yi_final,

V = vi,

random = ~ 1 | id/uid,

data = BSvsPLA_short,

)

meta_BSvsPLA_overallefficacy_sexmod_short<-rma.mv(yi = yi_final,

V = vi,

random = ~ 1 | id/uid,

data = BSvsPLA_short,

mods = ~sex)

meta_BSvsPLA_frequency_main_short<-rma.mv(yi = yi_final,

V = vi,

random = ~ 1 | id/uid,

data = BSvsPLA_short,

subset = outcome_domain =="Frequency"

)

meta_BSvsPLA_frequency_sexmod_short<-rma.mv(yi = yi_final,

V = vi,

random = ~ 1 | id/uid,

data = BSvsPLA_short,

subset = outcome_domain =="Frequency",

mods = ~sex)

meta_BSvsPLA_quantity_main_short<-rma.mv(yi = yi_final,

V = vi,

random = ~ 1 | id/uid,

data = BSvsPLA_short,

subset = outcome_domain =="Quantity"

)

meta_BSvsPLA_quantity_sexmod_short<-rma.mv(yi = yi_final,

V = vi,

random = ~ 1 | id/uid,

data = BSvsPLA_short,

subset = outcome_domain =="Quantity",

mods = ~sex)

### NorthAmerica subgroup

meta_BSvsPLA_overallefficacy_main_NorthAmerica<-rma.mv(yi = yi_final,

V = vi,

random = ~ 1 | id/uid,

data = BSvsPLA_NorthAmerica,

)

meta_BSvsPLA_overallefficacy_sexmod_NorthAmerica<-rma.mv(yi = yi_final,

V = vi,

random = ~ 1 | id/uid,

data = BSvsPLA_NorthAmerica,

mods = ~sex)

meta_BSvsPLA_frequency_main_NorthAmerica<-rma.mv(yi = yi_final,

V = vi,

random = ~ 1 | id/uid,

data = BSvsPLA_NorthAmerica,

subset = outcome_domain =="Frequency"

)

meta_BSvsPLA_frequency_sexmod_NorthAmerica<-rma.mv(yi = yi_final,

V = vi,

random = ~ 1 | id/uid,

data = BSvsPLA_NorthAmerica,

subset = outcome_domain =="Frequency",

mods = ~sex)

meta_BSvsPLA_quantity_main_NorthAmerica<-rma.mv(yi = yi_final,

V = vi,

random = ~ 1 | id/uid,

data = BSvsPLA_NorthAmerica,

subset = outcome_domain =="Quantity"

)

meta_BSvsPLA_quantity_sexmod_NorthAmerica<-rma.mv(yi = yi_final,

V = vi,

random = ~ 1 | id/uid,

data = BSvsPLA_NorthAmerica,

subset = outcome_domain =="Quantity",

mods = ~sex)

### HighROB subgroup

meta_BSvsPLA_overallefficacy_main_HighROB<-rma.mv(yi = yi_final,

V = vi,

random = ~ 1 | id/uid,

data = BSvsPLA_HighROB,

)

meta_BSvsPLA_overallefficacy_sexmod_HighROB<-rma.mv(yi = yi_final,

V = vi,

random = ~ 1 | id/uid,

data = BSvsPLA_HighROB,

mods = ~sex)

meta_BSvsPLA_frequency_main_HighROB<-rma.mv(yi = yi_final,

V = vi,

random = ~ 1 | id/uid,

data = BSvsPLA_HighROB,

subset = outcome_domain =="Frequency"

)

meta_BSvsPLA_frequency_sexmod_HighROB<-rma.mv(yi = yi_final,

V = vi,

random = ~ 1 | id/uid,

data = BSvsPLA_HighROB,

subset = outcome_domain =="Frequency",

mods = ~sex)

meta_BSvsPLA_quantity_main_HighROB<-rma.mv(yi = yi_final,

V = vi,

random = ~ 1 | id/uid,

data = BSvsPLA_HighROB,

subset = outcome_domain =="Quantity"

)

meta_BSvsPLA_quantity_sexmod_HighROB<-rma.mv(yi = yi_final,

V = vi,

random = ~ 1 | id/uid,

data = BSvsPLA_HighROB,

subset = outcome_domain =="Quantity",

mods = ~sex)

### LowMedRob subgroup

meta_BSvsPLA_overallefficacy_main_LowMedRob<-rma.mv(yi = yi_final,

V = vi,

random = ~ 1 | id/uid,

data = BSvsPLA_LowMedRob,

)

meta_BSvsPLA_overallefficacy_sexmod_LowMedRob<-rma.mv(yi = yi_final,

V = vi,

random = ~ 1 | id/uid,

data = BSvsPLA_LowMedRob,

mods = ~sex)

meta_BSvsPLA_frequency_main_LowMedRob<-rma.mv(yi = yi_final,

V = vi,

random = ~ 1 | id/uid,

data = BSvsPLA_LowMedRob,

subset = outcome_domain =="Frequency"

)

meta_BSvsPLA_frequency_sexmod_LowMedRob<-rma.mv(yi = yi_final,

V = vi,

random = ~ 1 | id/uid,

data = BSvsPLA_LowMedRob,

subset = outcome_domain =="Frequency",

mods = ~sex)

meta_BSvsPLA_quantity_main_LowMedRob<-rma.mv(yi = yi_final,

V = vi,

random = ~ 1 | id/uid,

data = BSvsPLA_LowMedRob,

subset = outcome_domain =="Quantity"

)

meta_BSvsPLA_quantity_sexmod_LowMedRob<-rma.mv(yi = yi_final,

V = vi,

random = ~ 1 | id/uid,

data = BSvsPLA_LowMedRob,

subset = outcome_domain =="Quantity",

mods = ~sex)

### YComorbid subgroup

meta_BSvsPLA_overallefficacy_main_YComorbid<-rma.mv(yi = yi_final,

V = vi,

random = ~ 1 | id/uid,

data = BSvsPLA_YComorbid,

)

meta_BSvsPLA_overallefficacy_sexmod_YComorbid<-rma.mv(yi = yi_final,

V = vi,

random = ~ 1 | id/uid,

data = BSvsPLA_YComorbid,

mods = ~sex)

meta_BSvsPLA_frequency_main_YComorbid<-rma.mv(yi = yi_final,

V = vi,

random = ~ 1 | id/uid,

data = BSvsPLA_YComorbid,

subset = outcome_domain =="Frequency"

)

meta_BSvsPLA_frequency_sexmod_YComorbid<-rma.mv(yi = yi_final,

V = vi,

random = ~ 1 | id/uid,

data = BSvsPLA_YComorbid,

subset = outcome_domain =="Frequency",

mods = ~sex)

meta_BSvsPLA_quantity_main_YComorbid<-rma.mv(yi = yi_final,

V = vi,

random = ~ 1 | id/uid,

data = BSvsPLA_YComorbid,

subset = outcome_domain =="Quantity"

)

meta_BSvsPLA_quantity_sexmod_YComorbid<-rma.mv(yi = yi_final,

V = vi,

random = ~ 1 | id/uid,

data = BSvsPLA_YComorbid,

subset = outcome_domain =="Quantity",

mods = ~sex)

### NComorbid subgroup

meta_BSvsPLA_overallefficacy_main_NComorbid<-rma.mv(yi = yi_final,

V = vi,

random = ~ 1 | id/uid,

data = BSvsPLA_NComorbid,

)

meta_BSvsPLA_overallefficacy_sexmod_NComorbid<-rma.mv(yi = yi_final,

V = vi,

random = ~ 1 | id/uid,

data = BSvsPLA_NComorbid,

mods = ~sex)

meta_BSvsPLA_frequency_main_NComorbid<-rma.mv(yi = yi_final,

V = vi,

random = ~ 1 | id/uid,

data = BSvsPLA_NComorbid,

subset = outcome_domain =="Frequency"

)

meta_BSvsPLA_frequency_sexmod_NComorbid<-rma.mv(yi = yi_final,

V = vi,

random = ~ 1 | id/uid,

data = BSvsPLA_NComorbid,

subset = outcome_domain =="Frequency",

mods = ~sex)

meta_BSvsPLA_quantity_main_NComorbid<-rma.mv(yi = yi_final,

V = vi,

random = ~ 1 | id/uid,

data = BSvsPLA_NComorbid,

subset = outcome_domain =="Quantity"

)

meta_BSvsPLA_quantity_sexmod_NComorbid<-rma.mv(yi = yi_final,

V = vi,

random = ~ 1 | id/uid,

data = BSvsPLA_NComorbid,

subset = outcome_domain =="Quantity",

mods = ~sex)

summary(meta_BSvsPLA_overallefficacy_main_opioid)

summary(meta_BSvsPLA_overallefficacy_sexmod_opioid)

summary(meta_BSvsPLA_frequency_main_opioid)

summary(meta_BSvsPLA_frequency_sexmod_opioid)

summary(meta_BSvsPLA_quantity_main_opioid)

summary(meta_BSvsPLA_quantity_sexmod_opioid)

summary(meta_BSvsPLA_overallefficacy_main_GABA)

summary(meta_BSvsPLA_overallefficacy_sexmod_GABA)

summary(meta_BSvsPLA_frequency_main_GABA)

summary(meta_BSvsPLA_frequency_sexmod_GABA)

summary(meta_BSvsPLA_overallefficacy_main_bDrinking)

summary(meta_BSvsPLA_overallefficacy_sexmod_bDrinking)

summary(meta_BSvsPLA_frequency_main_bDrinking)

summary(meta_BSvsPLA_frequency_sexmod_bDrinking)

summary(meta_BSvsPLA_quantity_main_bDrinking)

summary(meta_BSvsPLA_quantity_sexmod_bDrinking)

summary(meta_BSvsPLA_overallefficacy_main_short)

summary(meta_BSvsPLA_overallefficacy_sexmod_short)

summary(meta_BSvsPLA_frequency_main_short)

summary(meta_BSvsPLA_frequency_sexmod_short)

summary(meta_BSvsPLA_quantity_main_short)

summary(meta_BSvsPLA_quantity_sexmod_short)

summary(meta_BSvsPLA_overallefficacy_main_NorthAmerica)

summary(meta_BSvsPLA_overallefficacy_sexmod_NorthAmerica)

summary(meta_BSvsPLA_frequency_main_NorthAmerica)

summary(meta_BSvsPLA_frequency_sexmod_NorthAmerica)

summary(meta_BSvsPLA_quantity_main_NorthAmerica)

summary(meta_BSvsPLA_quantity_sexmod_NorthAmerica)

summary(meta_BSvsPLA_overallefficacy_main_HighROB)

summary(meta_BSvsPLA_overallefficacy_sexmod_HighROB)

summary(meta_BSvsPLA_frequency_main_HighROB)

summary(meta_BSvsPLA_frequency_sexmod_HighROB)

summary(meta_BSvsPLA_quantity_main_HighROB)

summary(meta_BSvsPLA_quantity_sexmod_HighROB)

summary(meta_BSvsPLA_overallefficacy_main_LowMedRob)

summary(meta_BSvsPLA_overallefficacy_sexmod_LowMedRob)

summary(meta_BSvsPLA_frequency_main_LowMedRob)

summary(meta_BSvsPLA_frequency_sexmod_LowMedRob)

summary(meta_BSvsPLA_quantity_main_LowMedRob)

summary(meta_BSvsPLA_quantity_sexmod_LowMedRob)

summary(meta_BSvsPLA_overallefficacy_main_YComorbid)

summary(meta_BSvsPLA_overallefficacy_sexmod_YComorbid)

summary(meta_BSvsPLA_frequency_main_YComorbid)

summary(meta_BSvsPLA_frequency_sexmod_YComorbid)

summary(meta_BSvsPLA_quantity_main_YComorbid)

summary(meta_BSvsPLA_quantity_sexmod_YComorbid)

summary(meta_BSvsPLA_overallefficacy_main_NComorbid)

summary(meta_BSvsPLA_overallefficacy_sexmod_NComorbid)

summary(meta_BSvsPLA_frequency_main_NComorbid)

summary(meta_BSvsPLA_frequency_sexmod_NComorbid)

summary(meta_BSvsPLA_quantity_main_NComorbid)

summary(meta_BSvsPLA_quantity_sexmod_NComorbid)

mlm.variance.distribution(meta_BSvsPLA_overallefficacy_main_opioid)

mlm.variance.distribution(meta_BSvsPLA_frequency_main_opioid)

mlm.variance.distribution(meta_BSvsPLA_quantity_main_opioid)

mlm.variance.distribution(meta_BSvsPLA_overallefficacy_main_GABA)

mlm.variance.distribution(meta_BSvsPLA_frequency_main_GABA)

mlm.variance.distribution(meta_BSvsPLA_quantity_main_GABA)

mlm.variance.distribution(meta_BSvsPLA_overallefficacy_main_bDrinking)

mlm.variance.distribution(meta_BSvsPLA_frequency_main_bDrinking)

mlm.variance.distribution(meta_BSvsPLA_quantity_main_bDrinking)

mlm.variance.distribution(meta_BSvsPLA_overallefficacy_main_short)

mlm.variance.distribution(meta_BSvsPLA_frequency_main_short)

mlm.variance.distribution(meta_BSvsPLA_quantity_main_short)

mlm.variance.distribution(meta_BSvsPLA_overallefficacy_main_NorthAmerica)

mlm.variance.distribution(meta_BSvsPLA_frequency_main_NorthAmerica)

mlm.variance.distribution(meta_BSvsPLA_quantity_main_NorthAmerica)

mlm.variance.distribution(meta_BSvsPLA_overallefficacy_main_HighROB)

mlm.variance.distribution(meta_BSvsPLA_frequency_main_HighROB)

mlm.variance.distribution(meta_BSvsPLA_quantity_main_HighROB)

mlm.variance.distribution(meta_BSvsPLA_overallefficacy_main_LowMedRob)

mlm.variance.distribution(meta_BSvsPLA_frequency_main_LowMedRob)

mlm.variance.distribution(meta_BSvsPLA_quantity_main_LowMedRob)

mlm.variance.distribution(meta_BSvsPLA_overallefficacy_main_YComorbid)

mlm.variance.distribution(meta_BSvsPLA_frequency_main_YComorbid)

mlm.variance.distribution(meta_BSvsPLA_quantity_main_YComorbid)

mlm.variance.distribution(meta_BSvsPLA_overallefficacy_main_NComorbid)

mlm.variance.distribution(meta_BSvsPLA_frequency_main_NComorbid)

mlm.variance.distribution(meta_BSvsPLA_quantity_main_NComorbid)

################################## MODERATOR ANALYSES

BSvsPLA_baseline <- subset(BSvsPLA, control_baseline == "Yes")

### check number of trials for moderator analysis eligiblity

table(!is.na(BSvsPLA$tx_length_weeks))

table(!is.na(BSvsPLA$age_mean1))

length(unique(subset(BSvsPLA, !is.na(tx_length_weeks))$id))

length(unique(subset(BSvsPLA, !is.na(age_mean1))$id))

length(unique(BSvsPLA_baseline$id))

BSvsPLA$baseline_mean <- rowMeans(cbind(BSvsPLA$b_mean1_32, BSvsPLA$b_mean1_33), na.rm = TRUE)

#Treatment Length (continuous)

meta_txlength_overall <- rma.mv(

yi = yi_final,

V = vi,

random = ~1 | id/uid,

data = BSvsPLA,

mods = ~ tx_length_weeks

)

meta_txlength_freq <- rma.mv(

yi = yi_final,

V = vi,

random = ~1 | id/uid,

data = BSvsPLA_freq,

mods = ~ tx_length_weeks

)

meta_txlength_quant <- rma.mv(

yi = yi_final,

V = vi,

random = ~1 | id/uid,

data = BSvsPLA_quant,

mods = ~ tx_length_weeks

)

meta_sex_txlength_overall <- rma.mv(

yi = yi_final,

V = vi,

random = ~1 | id/uid,

data = BSvsPLA,

mods = ~ sex * tx_length_weeks

)

meta_sex_txlength_freq <- rma.mv(

yi = yi_final,

V = vi,

random = ~1 | id/uid,

data = BSvsPLA_freq,

mods = ~ sex * tx_length_weeks

)

meta_sex_txlength_quant <- rma.mv(

yi = yi_final,

V = vi,

random = ~1 | id/uid,

data = BSvsPLA_quant,

mods = ~ sex * tx_length_weeks

)

# Age

meta_age_overall <- rma.mv(

yi = yi_final,

V = vi,

random = ~1 | id/uid,

data = BSvsPLA,

mods = ~ sample_age

)

meta_age_freq <- rma.mv(

yi = yi_final,

V = vi,

random = ~1 | id/uid,

data = BSvsPLA_freq,

mods = ~ sample_age

)

meta_age_quant <- rma.mv(

yi = yi_final,

V = vi,

random = ~1 | id/uid,

data = BSvsPLA_quant,

mods = ~ sample_age

)

meta_sex_age_overall <- rma.mv(

yi = yi_final,

V = vi,

random = ~1 | id/uid,

data = BSvsPLA,

mods = ~ sex * sample_age

)

meta_sex_age_freq <- rma.mv(

yi = yi_final,

V = vi,

random = ~1 | id/uid,

data = BSvsPLA_freq,

mods = ~ sex * sample_age

)

meta_sex_age_quant <- rma.mv(

yi = yi_final,

V = vi,

random = ~1 | id/uid,

data = BSvsPLA_quant,

mods = ~ sex * sample_age

)

summary(meta_txlength_overall)

summary(meta_txlength_freq)

summary(meta_sex_txlength_overall)

summary(meta_sex_txlength_freq)

summary(meta_age_overall)

summary(meta_age_freq)

summary(meta_sex_age_overall)

summary(meta_sex_age_freq)

# baseline

meta_baseline_overall <- rma.mv(

yi = yi_final,

V = vi,

random = ~1 | id/uid,

data = BSvsPLA,

mods = ~ baseline_mean

)

meta_sex_baseline_overall <- rma.mv(

yi = yi_final,

V = vi,

random = ~1 | id/uid,

data = BSvsPLA,

mods = ~ sex * baseline_mean

)

summary(meta_baseline_overall)

summary(meta_sex_baseline_overall)

mlm.variance.distribution(meta_baseline_overall)

mlm.variance.distribution(meta_age_overall)

mlm.variance.distribution(meta_age_freq)

mlm.variance.distribution(meta_txlength_overall)

mlm.variance.distribution(meta_txlength_freq)

################## BINARY DTA

BI<-subset(data_file, data_file$summary_statistic=="Risk Ratio")

BI<-escalc(measure = "RR",

ai = event1_n,

n1i = n1,

ci = event2_n,

n2i = n2,

data = BI)

summary(BI)

BI$uid<-1:nrow(BI)

meta_BI_OE_main<-rma.mv(yi = yi,

V = vi,

random = ~ 1 | id/uid,

data = BI,

)

meta_BI_OE_sex<-rma.mv(yi = yi,

V = vi,

random = ~ 1 | id/uid,

data = BI,

mods = ~sex,

)

summary(meta_BI_OE_main)

exp( -0.0465) #exponentiate the estimate

exp(-0.6472) #exponentiate low CI

exp(0.5542) #exponentiate upper CI

summary(meta_BI_OE_sex)

exp(0.0406)

exp(-0.1418)

exp(0.2230)

length(unique(BI$uid))

length(unique(BI$id))

mlm.variance.distribution(meta_BI_OE_main)

########################### Forest plots

BSvsPLA$slab_label <- paste0(BSvsPLA$ref, ": ", BSvsPLA$uid_label, " - ", BSvsPLA$baby_domain, " (", BSvsPLA$sex, ")")

tiff("forest_BSvsPLA_OE_main_3.tiff", width = 3600, height = 2900, res = 300, compression = "lzw")

forest(meta_BSvsPLA_OE_main,

slab = BSvsPLA$slab_label,

xlab = "Standardised Mean Difference (SMD)",

alim = c(-4, 4),

at = seq(-4, 4, 1),

cex = 0.75)

dev.off()

within$slab_label <- paste0(

within$ref, ": ", within$uid_label_3," - ", within$outcome, " - ", within$sex)

forest(meta_WS_OE_main,

slab = within$slab_label,

xlab = "Standardized Mean Difference (SMD)",

mlab = "Random-effects model",

alim = c(-6,6),

cex = 0.8,

psize = 0.8)

title("Pharmacotherapy Effectiveness on Overall Consumption Outcomes (within-subject)")

tiff("WS OE MAY 2025.tiff", width = 16, height = 12, units = "in", res = 300)

forest(meta_WS_OE_main,

slab = within$slab_label,

xlab = "Standardised Mean Difference (SMD)",

mlab = "Random-effects model",

alim = c(-6,6),

cex = 0.8,

psize = 0.8)

title("Pharmacotherapy Effectiveness on Overall Consumption Outcomes (within-subject)")

dev.off()

BI$slab_label <- paste0(

BI$ref, ": ", BI$uid_label_3," - ", BI$outcome, " - ", BI$sex)

forest(meta_BI_OE_main,

slab = BI$slab_label,

xlab = "Risk Ratio (RR)",

mlab = "Random-effects model",

alim = c(0.01, 10),

transf = exp,

cex = 0.8,

psize = 0.8)

tiff("BI MAY 2025.tiff", width = 16, height = 12, units = "in", res = 300)

forest(meta_BI_OE_main,

slab = BI$slab_label,

xlab = "Risk Ratio (RR)",

mlab = "Random-effects model",

alim = c(0.01, 10),

transf = exp,

cex = 0.8,

psize = 0.8)

title("Pharmacotherapy Effectiveness on Binary Outcomes")

dev.off()

######################## publication bias

## main effect funnel

funnel(meta_BSvsPLA_OE_main)

funnel(meta_BSvsPLA_OE_main,

xlab = "Effect Size (SMD)",

ylab = "Standard Error",

main = "Funnel Plot for Publication Bias (MLM; between-subjects, vs. control)")

#aggregate es per study to run single-level meta for eggers test eligibility

BSvsPLA_agg <- BSvsPLA %>%

group_by(id) %>%

summarise(

yi = sum(yi_final / vi) / sum(1 / vi),

vi = 1 / sum(1 / vi),

k = n()

)

#slm

meta_BSvsPLA_agg <- rma.uni(yi, vi, data = BSvsPLA_agg)

#eggers

regtest(meta_BSvsPLA_agg, model = "rma")

#slm funnel

funnel(meta_BSvsPLA_agg,

xlab = "Effect Size (SMD)",

ylab = "Standard Error",

main = "Funnel Plot for Publication Bias (SLM; between-subects, vs. control)")

BSvsPLA_OE_female <- subset(BSvsPLA, sex=="Female")

BSvsPLA_OE_male <- subset(BSvsPLA, sex=="Male")

## female meta

meta_BSvsPLA_OE_female<-rma.mv(yi = yi_final,

V = vi,

random = ~ 1 | id/uid,

data = BSvsPLA_OE_female,

)

summary(meta_BSvsPLA_OE_female)

mlm.variance.distribution(meta_BSvsPLA_OE_female)

## male meta

meta_BSvsPLA_OE_male<-rma.mv(yi = yi_final,

V = vi,

random = ~ 1 | id/uid,

data = BSvsPLA_OE_male,

)

summary(meta_BSvsPLA_OE_male)

mlm.variance.distribution(meta_BSvsPLA_OE_male)

funnel(meta_BSvsPLA_OE_female,

xlab = "Effect Size (SMD)",

ylab = "Standard Error",

main = "Funnel Plot for Publication Bias - Female Subjects Only (MLM; between-subjects, vs. control)")

funnel(meta_BSvsPLA_OE_male,

xlab = "Effect Size (SMD)",

ylab = "Standard Error",

main = "Funnel Plot for Publication Bias - Male Subjects Only (MLM; between-subjects, vs. control)")

## Female aggregation

BSvsPLA_female_agg <- BSvsPLA_OE_female %>%

group_by(id) %>%

summarise(

yi = sum(yi_final / vi) / sum(1 / vi),

vi = 1 / sum(1 / vi),

k = n()

)

## Male aggregation

BSvsPLA_male_agg <- BSvsPLA_OE_male %>%

group_by(id) %>%

summarise(

yi = sum(yi_final / vi) / sum(1 / vi),

vi = 1 / sum(1 / vi),

k = n()

)

## Female SLM

meta_BSvsPLA_female_agg <- rma.uni(yi, vi, data = BSvsPLA_female_agg)

## Male SLM

meta_BSvsPLA_male_agg <- rma.uni(yi, vi, data = BSvsPLA_male_agg)

regtest(meta_BSvsPLA_female_agg, model = "rma")

regtest(meta_BSvsPLA_male_agg, model = "rma")

## Funnel Female SLM

funnel(meta_BSvsPLA_female_agg,

xlab = "Effect Size (SMD)",

ylab = "Standard Error",

main = "Funnel Plot for Publication Bias - Female Subjects Only (SLM)")

## Funnel Male SLM

funnel(meta_BSvsPLA_male_agg,

xlab = "Effect Size (SMD)",

ylab = "Standard Error",

main = "Funnel Plot for Publication Bias - Male Subjects Only (SLM)")
