# Supplementary material for: Effectiveness of Alcohol Use Disorder Pharmacotherapies by Sex: Systematic Review and Meta‐Analysis
Source: Drug Alcohol Rev. 2026 Jun 23;45(5):e70196. doi: 10.1111/dar.70196 (PMC13290497; doi:10.1111/dar.70196)
Supplement: Supplementary file 8 — Table S1: Search strategy terms. [file DAR-45-0-s007.docx]

| **Table S1. Search Strategy Terms** | |
| --- | --- |
| Population Terms | alcohol* OR ethanol OR alcohol use disorder* |
| Intervention Terms | acamprosate OR amisulpride OR aripiprazole OR atenolol OR baclofen OR carbamazepine OR citalopram OR escitalopram OR disulfiram OR fluoxetine OR flupenthixol OR fluvoxamine OR gabapentin OR galantamine OR gamma-hydroxybutyric acid OR GHB OR levetiracetam OR lisuride OR lithium OR memantine OR modafinil OR nalmefene OR naltrexone OR nefazodone OR ondansetron OR oxcarbazepine OR pregabalin OR quetiapine OR rimonabant OR tianeptine OR tiapride OR topiramate OR trazodone OR varenicline |
| Treatment Terms | treat* OR patient* OR service use* |

| **Search Strategy for Each Database** | |
| --- | --- |
| **Web of Science** | (TS=(alcohol* OR ethanol OR "alcohol use disorder*")) AND (TS=(treat* OR patient* OR "service use*")) AND (TS=(acamprosate OR amisulpride OR aripiprazole OR atenolol OR baclofen OR carbamazepine OR citalopram OR escitalopram OR disulfiram OR fluoxetine OR flupenthixol OR fluvoxamine OR gabapentin OR galantamine OR "gamma-hydroxybutyric acid" OR GHB OR levetiracetam OR lisuride OR lithium OR memantine OR modafinil OR nalmefene OR naltrexone OR nefazodone OR ondansetron OR oxcarbazepine OR pregabalin OR quetiapine OR rimonabant OR tianeptine OR tiapride OR topiramate OR trazodone OR varenicline)) |
| **PubMed** | ((alcohol*[tiab] OR ethanol[tiab] OR "alcohol use disorder*"[tiab])) AND ((treat*[tiab] OR patient*[tiab] OR "service use*"[tiab])) AND ((acamprosate[tiab] OR amisulpride[tiab] OR aripiprazole[tiab] OR atenolol[tiab] OR baclofen[tiab] OR carbamazepine[tiab] OR citalopram[tiab] OR escitalopram[tiab] OR disulfiram[tiab] OR fluoxetine[tiab] OR flupenthixol[tiab] OR fluvoxamine[tiab] OR gabapentin[tiab] OR galantamine[tiab] OR "gamma-hydroxybutyric acid"[tiab] OR GHB[tiab] OR levetiracetam[tiab] OR lisuride[tiab] OR lithium[tiab] OR memantine[tiab] OR modafinil[tiab] OR nalmefene[tiab] OR naltrexone[tiab] OR nefazodone[tiab] OR ondansetron[tiab] OR oxcarbazepine[tiab] OR pregabalin[tiab] OR quetiapine[tiab] OR rimonabant[tiab] OR tianeptine[tiab] OR tiapride[tiab] OR topiramate[tiab] OR trazodone[tiab] OR varenicline[tiab])) |
| **Scopus** | (( TITLE-ABS-KEY ( alcohol* ) OR TITLE-ABS-KEY ( ethanol ) OR TITLE-ABS-KEY ( alcohol AND use AND disorder* ) )) AND (( TITLE-ABS-KEY ( treat* ) OR TITLE-ABS-KEY ( patient* ) OR TITLE-ABS-KEY ( service AND use* ) )) AND (( TITLE-ABS-KEY ( acamprosate ) OR TITLE-ABS-KEY ( amisulpride ) OR TITLE-ABS-KEY ( aripiprazole ) OR TITLE-ABS-KEY ( atenolol ) OR TITLE-ABS-KEY ( baclofen ) OR TITLE-ABS-KEY ( carbamazepine ) OR TITLE-ABS-KEY ( citalopram ) OR TITLE-ABS-KEY ( escitalopram ) OR TITLE-ABS-KEY ( disulfiram ) OR TITLE-ABS-KEY ( fluoxetine ) OR TITLE-ABS-KEY ( flupenthixol ) OR TITLE-ABS-KEY ( fluvoxamine ) OR TITLE-ABS-KEY ( gabapentin ) OR TITLE-ABS-KEY ( galantamine ) OR TITLE-ABS-KEY ( "gamma-hydroxybutyric acid" ) OR TITLE-ABS-KEY ( "GHB" ) OR TITLE-ABS-KEY ( levetiracetam ) OR TITLE-ABS-KEY ( lisuride ) OR TITLE-ABS-KEY ( lithium ) OR TITLE-ABS-KEY ( memantine ) OR TITLE-ABS-KEY ( modafinil ) OR TITLE-ABS-KEY ( nalmefene ) OR TITLE-ABS-KEY ( naltrexone ) OR TITLE-ABS-KEY ( nefazodone ) OR TITLE-ABS-KEY ( ondansetron ) OR TITLE-ABS-KEY ( oxcarbazepine ) OR TITLE-ABS-KEY ( pregabalin ) OR TITLE-ABS-KEY ( quetiapine ) OR TITLE-ABS-KEY ( rimonabant ) OR TITLE-ABS-KEY ( tianeptine ) OR TITLE-ABS-KEY ( tiapride ) OR TITLE-ABS-KEY ( topiramate ) OR TITLE-ABS-KEY ( trazodone ) OR TITLE-ABS-KEY ( varenicline ) )) |
| **PsycINFO** | ((title: (alcohol*) *OR* title: (ethanol) *OR* title: ("alcohol use disorder*")) *OR* (abstract: (alcohol*) *OR* abstract: (ethanol) *OR* abstract: ("alcohol use disorder*")) *OR* (Keywords: (alcohol*) *OR* Keywords: (ethanol) *OR* Keywords: ("alcohol use disorder*"))) *AND* ((title: (treat*) *OR* title: (patient*) *OR* title: ("service use*")) *OR* (abstract: (treat*) *OR* abstract: (patient*) *OR* abstract: ("service use*")) *OR* (Keywords: (treat*) *OR* Keywords: (patient*) *OR* Keywords: ("service use*"))) *AND* ((title: (acamprosate) *OR* title: (amisulpride) *OR* title: (aripiprazole) *OR* title: (atenolol) *OR* title: (baclofen) *OR* title: (carbamazepine) *OR* title: (citalopram) *OR* title: (escitalopram) *OR* title: (disulfiram) *OR* title: (fluoxetine) *OR* title: (flupenthixol) *OR* title: (fluvoxamine) *OR* title: (gabapentin) *OR* title: (galantamine) *OR* title: ("gamma-hydroxybutyric acid") *OR* title: (GHB) *OR* title: (levetiracetam) *OR* title: (lisuride) *OR* title: (lithium) *OR* title: (memantine) *OR* title: (modafinil) *OR* title: (nalmefene) *OR* title: (naltrexone) *OR* title: (nefazodone) *OR* title: (ondansetron) *OR* title: (oxcarbazepine) *OR* title: (pregabalin) *OR* title: (quetiapine) *OR* title: (rimonabant) *OR* title: (tianeptine) *OR* title: (tiapride) *OR* title: (topiramate) *OR* title: (trazodone) *OR* title: (varenicline)) *OR* (abstract: (acamprosate) *OR* abstract: (amisulpride) *OR* abstract: (aripiprazole) *OR* abstract: (atenolol) *OR* abstract: (baclofen) *OR* abstract: (carbamazepine) *OR* abstract: (citalopram) *OR* abstract: (escitalopram) *OR* abstract: (disulfiram) *OR* abstract: (fluoxetine) *OR* abstract: (flupenthixol) *OR* abstract: (fluvoxamine) *OR* abstract: (gabapentin) *OR* abstract: (galantamine) *OR* abstract: ("gamma-hydroxybutyric acid") *OR* abstract: (GHB) *OR* abstract: (levetiracetam) *OR* abstract: (lisuride) *OR* abstract: (lithium) *OR* abstract: (memantine) *OR* abstract: (modafinil) *OR* abstract: (nalmefene) *OR* abstract: (naltrexone) *OR* abstract: (nefazodone) *OR* abstract: (ondansetron) *OR* abstract: (oxcarbazepine) *OR* abstract: (pregabalin) *OR* abstract: (quetiapine) *OR* abstract: (rimonabant) *OR* abstract: (tianeptine) *OR* abstract: (tiapride) *OR* abstract: (topiramate) *OR* abstract: (trazodone) *OR* abstract: (varenicline)) *OR* (Keywords: (acamprosate) *OR* Keywords: (amisulpride) *OR* Keywords: (aripiprazole) *OR* Keywords: (atenolol) *OR* Keywords: (baclofen) *OR* Keywords: (carbamazepine) *OR* Keywords: (citalopram) *OR* Keywords: (escitalopram) *OR* Keywords: (disulfiram) *OR* Keywords: (fluoxetine) *OR* Keywords: (flupenthixol) *OR* Keywords: (fluvoxamine) *OR* Keywords: (gabapentin) *OR* Keywords: (galantamine) *OR* Keywords: ("gamma-hydroxybutyric acid") *OR* Keywords: (GHB) *OR* Keywords: (levetiracetam) *OR* Keywords: (lisuride) *OR* Keywords: (lithium) *OR* Keywords: (memantine) *OR* Keywords: (modafinil) *OR* Keywords: (nalmefene) *OR* Keywords: (naltrexone) *OR* Keywords: (nefazodone) *OR* Keywords: (ondansetron) *OR* Keywords: (oxcarbazepine) *OR* Keywords: (pregabalin) *OR* Keywords: (quetiapine) *OR* Keywords: (rimonabant) *OR* Keywords: (tianeptine) *OR* Keywords: (tiapride) *OR* Keywords: (topiramate) *OR* Keywords: (trazodone) *OR* Keywords: (varenicline))) |
| **Cochrane** | ((alcohol* OR ethanol OR (alcohol NEXT use NEXT disorder*)):ti,ab,kw) AND ((treat* OR patient* OR (service NEXT use*)):ti,ab,kw) AND ((acamprosate OR amisulpride OR aripiprazole OR atenolol OR baclofen OR carbamazepine OR citalopram OR escitalopram OR disulfiram OR fluoxetine OR flupenthixol OR fluvoxamine OR gabapentin OR galantamine OR (gamma-hydroxybutyric NEXT acid) OR GHB OR levetiracetam OR lisuride OR lithium OR memantine OR modafinil OR nalmefene OR naltrexone OR nefazodone OR ondansetron OR oxcarbazepine OR pregabalin OR quetiapine OR rimonabant OR tianeptine OR tiapride OR topiramate OR trazodone OR varenicline):ti,ab,kw) |
