# Supplementary material for: Effectiveness of Alcohol Use Disorder Pharmacotherapies by Sex: Systematic Review and Meta‐Analysis
Source: Drug Alcohol Rev. 2026 Jun 23;45(5):e70196. doi: 10.1111/dar.70196 (PMC13290497; doi:10.1111/dar.70196)
Supplement: Supplementary file 9 — Table S2: Summary of data conversions applied to extracted estimates. [file DAR-45-0-s005.docx]

| **Table S2. Summary of Data Conversions Applied to Extracted Estimates** | | |
| --- | --- | --- |
| **Reference** | **Conversion** | **Method** |
| Garbutt et al. (2005) | Median & IQ Range -> *Mean* | Mean = (first quartile + median + third quartile)/3  *Scenario 3 from Wan et al. (2014) using an excel conversion sheet* |
| Garbutt et al. (2005) | Median & IQ Range -> *SD* | SD = (third quartile - first quartile)/η(n)  *Scenario 3 from Wan et al. (2014) using an excel conversion sheet* |
| Morley et al. (2022) | SEM -> *SD* | SD = SEM/√N |
| Naranjo et al. (2000) | SEM -> *SD* | SD = SEM/√N |
| O'Malley et al. (2018) | Median, IQ Range, min/max values -> *SD* | SD = (min value + 2(first quartile) + 2(median) + 2(third quartile) + max value)/8  *Scenario 2 from Wan et al. (2014) using an excel conversion sheet* |
| Yoon et al. (2016) | SEM -> *SD* | SD = SEM/√N |
