# Supplementary material for: Effectiveness of Alcohol Use Disorder Pharmacotherapies by Sex: Systematic Review and Meta‐Analysis
Source: Drug Alcohol Rev. 2026 Jun 23;45(5):e70196. doi: 10.1111/dar.70196 (PMC13290497; doi:10.1111/dar.70196)
Supplement: Supplementary file 10 — Table S3: PRISMA checklist. [file DAR-45-0-s012.docx]

**Table S3. PRISMA Checklist**

| **Section and Topic** | **Item #** | **Checklist item** | **Location where item is reported** |
| --- | --- | --- | --- |
| **TITLE** | | |  |
| Title | 1 | Identify the report as a systematic review. | Title page, line 3 |
| **ABSTRACT** | | |  |
| Abstract | 2 | See the PRISMA 2020 for Abstracts checklist. | Abstract written based on this checklist. Page 2, line 56 |
| **INTRODUCTION** | | |  |
| Rationale | 3 | Describe the rationale for the review in the context of existing knowledge. | Introduction: Page 3, lines 111-141 |
| Objectives | 4 | Provide an explicit statement of the objective(s) or question(s) the review addresses. | Page 3, lines 142-144 |
| **METHODS** | | |  |
| Eligibility criteria | 5 | Specify the inclusion and exclusion criteria for the review and how studies were grouped for the syntheses. | Study selection and eligibility criteria: page 5, lines x-y, and Table 1 |
| Information sources | 6 | Specify all databases, registers, websites, organisations, reference lists and other sources searched or consulted to identify studies. Specify the date when each source was last searched or consulted. | Page 4, lines 150-152 |
| Search strategy | 7 | Present the full search strategies for all databases, registers and websites, including any filters and limits used. | Supplementary materials table S1 |
| Selection process | 8 | Specify the methods used to decide whether a study met the inclusion criteria of the review, including how many reviewers screened each record and each report retrieved, whether they worked independently, and if applicable, details of automation tools used in the process. | Study selection and eligibility criteria: page 4, lines 165-181 |
| Data collection process | 9 | Specify the methods used to collect data from reports, including how many reviewers collected data from each report, whether they worked independently, any processes for obtaining or confirming data from study investigators, and if applicable, details of automation tools used in the process. | Data extraction: page 5, lines 186-193 |
| Data items | 10a | List and define all outcomes for which data were sought. Specify whether all results that were compatible with each outcome domain in each study were sought (e.g. for all measures, time points, analyses), and if not, the methods used to decide which results to collect. | Page 4, Table 1, footnote b & c |
|  | 10b | List and define all other variables for which data were sought (e.g. participant and intervention characteristics, funding sources). Describe any assumptions made about any missing or unclear information. | Data extraction: page 5, lines 186-193 |
| Study risk of bias assessment | 11 | Specify the methods used to assess risk of bias in the included studies, including details of the tool(s) used, how many reviewers assessed each study and whether they worked independently, and if applicable, details of automation tools used in the process. | Risk of bias: page 5, lines 196-205 |
| Effect measures | 12 | Specify for each outcome the effect measure(s) (e.g. risk ratio, mean difference) used in the synthesis or presentation of results. | Meta-analytic approach: page 5, lines 216-220, and page 6, lines 221-252 |
| Synthesis methods | 13a | Describe the processes used to decide which studies were eligible for each synthesis (e.g. tabulating the study intervention characteristics and comparing against the planned groups for each synthesis (item #5)). | Page 4, Table 1, footnote e |
|  | 13b | Describe any methods required to prepare the data for presentation or synthesis, such as handling of missing summary statistics, or data conversions. | Data extraction: page 5, lines 186-193  Supplementary materials, table S2 |
|  | 13c | Describe any methods used to tabulate or visually display results of individual studies and syntheses. | Narrative synthesis: page 5, lines 208-213  Supplementary materials figure S5 |
|  | 13d | Describe any methods used to synthesize results and provide a rationale for the choice(s). If meta-analysis was performed, describe the model(s), method(s) to identify the presence and extent of statistical heterogeneity, and software package(s) used. | Narrative synthesis: page 5, lines 208-213 |
|  | 13e | Describe any methods used to explore possible causes of heterogeneity among study results (e.g. subgroup analysis, meta-regression). | Meta-analytic approach: page 6, lines 234-241 |
|  | 13f | Describe any sensitivity analyses conducted to assess robustness of the synthesized results. | Subgroup analyses: page 6, lines 234-241 |
| Reporting bias assessment | 14 | Describe any methods used to assess risk of bias due to missing results in a synthesis (arising from reporting biases). | Publication bias: page 11, lines 394-400 |
| Certainty assessment | 15 | Describe any methods used to assess certainty (or confidence) in the body of evidence for an outcome. | Supplementary materials figure S6 |
| **RESULTS** | | |  |
| Study selection | 16a | Describe the results of the search and selection process, from the number of records identified in the search to the number of studies included in the review, ideally using a flow diagram. | Figure 1 PRISMA Flow: page 6. |
|  | 16b | Cite studies that might appear to meet the inclusion criteria, but which were excluded, and explain why they were excluded. | Supplementary Materials table SX. |
| Study characteristics | 17 | Cite each included study and present its characteristics. | Table 2: page 8, line 299 |
| Risk of bias in studies | 18 | Present assessments of risk of bias for each included study. | Supplementary materials figure S1. |
| Results of individual studies | 19 | For all outcomes, present, for each study: (a) summary statistics for each group (where appropriate) and (b) an effect estimate and its precision (e.g. confidence/credible interval), ideally using structured tables or plots. | Supplementary materials figures S2-S4. |
| Results of syntheses | 20a | For each synthesis, briefly summarise the characteristics and risk of bias among contributing studies. | Table 2: page 8, line 299 |
|  | 20b | Present results of all statistical syntheses conducted. If meta-analysis was done, present for each the summary estimate and its precision (e.g. confidence/credible interval) and measures of statistical heterogeneity. If comparing groups, describe the direction of the effect. | Tables 3-6: page 10, lines 369, 371, and 373 |
|  | 20c | Present results of all investigations of possible causes of heterogeneity among study results. | Subgroup and meta-regression analyses: page 9, lines 331-359 |
|  | 20d | Present results of all sensitivity analyses conducted to assess the robustness of the synthesized results. | Subgroup and meta-regression analyses: page 9, lines 331-359 |
| Reporting biases | 21 | Present assessments of risk of bias due to missing results (arising from reporting biases) for each synthesis assessed. | Supplementary materials figure SX |
| Certainty of evidence | 22 | Present assessments of certainty (or confidence) in the body of evidence for each outcome assessed. | Supplementary materials figure S6 |
| **DISCUSSION** | | |  |
| Discussion | 23a | Provide a general interpretation of the results in the context of other evidence. | Discussion: paragraphs 2,3, and 4. Pages 12-14, lines 473-516 |
|  | 23b | Discuss any limitations of the evidence included in the review. | Discussion paragraphs: pages 14 and 15, lines 530-557 |
|  | 23c | Discuss any limitations of the review processes used. | Page 15, lines 560-567 |
|  | 23d | Discuss implications of the results for practice, policy, and future research. | Pages 15 and 16, lines 569-592 |
| **OTHER INFORMATION** | | |  |
| Registration and protocol | 24a | Provide registration information for the review, including register name and registration number, or state that the review was not registered. | Protocol and registration: page 4, lines 148-150 |
|  | 24b | Indicate where the review protocol can be accessed, or state that a protocol was not prepared. | Protocol and registration: page 4, lines 148-150 |
|  | 24c | Describe and explain any amendments to information provided at registration or in the protocol. | None were made. |
| Support | 25 | Describe sources of financial or non-financial support for the review, and the role of the funders or sponsors in the review. | Title page |
| Competing interests | 26 | Declare any competing interests of review authors. | Title page |
| Availability of data, code and other materials | 27 | Report which of the following are publicly available and where they can be found: template data collection forms; data extracted from included studies; data used for all analyses; analytic code; any other materials used in the review. | Page 6, lines 252-253 |
|  |  |  |  |

*From:*  Page MJ, McKenzie JE, Bossuyt PM, Boutron I, Hoffmann TC, Mulrow CD, et al. The PRISMA 2020 statement: an updated guideline for reporting systematic reviews. BMJ 2021;372:n71. doi: 10.1136/bmj.n71. This work is licensed under CC BY 4.0. To view a copy of this license, visit <https://creativecommons.org/licenses/by/4.0/>
