# Supplementary material for: Effectiveness of Alcohol Use Disorder Pharmacotherapies by Sex: Systematic Review and Meta‐Analysis
Source: Drug Alcohol Rev. 2026 Jun 23;45(5):e70196. doi: 10.1111/dar.70196 (PMC13290497; doi:10.1111/dar.70196)
Supplement: Supplementary file 11 — Table S4: Moderator effects and sex × moderator interactions by multi‐level meta‐regression analyses. [file DAR-45-0-s004.docx]

| **Table S4. Moderator Effects and Sex × Moderator Interactions by Multi-Level Meta-Regression Analyses** | | | | | | | | | | | | | | | | | |
| --- | --- | --- | --- | --- | --- | --- | --- | --- | --- | --- | --- | --- | --- | --- | --- | --- | --- |
|  | | k/N trials | I² |  | **Moderator Effect** | | | | | |  | **Sex x Moderator Interaction Effect** | | | | | |
|  |  |  |  |  | β | SE | z | 95% CI | | *p* |  | β | SE | z | 95% CI | | *p* |
|  |  |  |  |  |  |  |  | LB | UB |  |  |  |  |  | LB | UB |  |
| **Baseline Severity** | |  |  |  |  |  |  |  |  |  |  |  |  |  |  |  |  |
|  | *Overall Efficacy* | 22/13 | 62.87 |  | -0.00 | 0.00 | -0.43 | -0.01 | 0.01 | 0.68 |  | 0.00 | 0.00 | 1.26 | -0.00 | 0.01 | 0.208 |
|  | *Frequency Outcomes* | 14/9 | - |  | - | - | - | - | - | - |  | - | - | - | - | - | - |
|  | *Quantity Outcomes* | 8/8 | - |  | - | - | - | - | - | - |  | - | - | - | - | - | - |
| **Sample Age** | |  |  |  |  |  |  |  |  |  |  |  |  |  |  |  |  |
|  | *Overall Efficacy* | 31/13 | 89.01 |  | 0.03 | 0.03 | 0.82 | -0.04 | 0.09 | 0.414 |  | -0.00 | 0.02 | -0.20 | -0.04 | 0.03 | 0.843 |
|  | *Frequency Outcomes* | 21/13 | 93.41 |  | 0.03 | 0.04 | 0.84 | -0.04 | 0.11 | 0.399 |  | -0.01 | 0.02 | -0.70 | -0.05 | 0.02 | 0.483 |
|  | *Quantity Outcomes* | 10/9 | - |  | - | - | - | - | - | - |  | - | - | - | - | - | - |
| **Treatment Length** | |  |  |  |  |  |  |  |  |  |  |  |  |  |  |  |  |
|  | *Overall Efficacy* | 26/11 | 79.64 |  | -0.00 | 0.04 | -0.11 | -0.08 | 0.07 | 0.916 |  | 0.02 | 0.03 | 0.61 | -0.04 | 0.08 | 0.543 |
|  | *Frequency Outcomes* | 16/11 | 88.09 |  | -0.00 | 0.05 | -0.06 | -0.10 | 0.09 | 0.951 |  | 0.02 | 0.05 | 0.42 | -0.07 | 0.11 | 0.672 |
|  | *Quantity Outcomes* | 10/9 | - |  | - | - | - | - | - | - |  | - | - | - | - | - | - |
| *Note.*  This table presents results from multilevel mixed-effects meta-regression models examining whether baseline severity, sample age, and treatment duration moderated pharmacotherapy effectiveness, and whether these effects differed by sex. All analyses were conducted using between-subject data comparing treatment to control conditions on measures of consumption reduction. Each moderator was first tested independently (*Moderator Effect*), followed by interaction models (*Sex × Moderator Interaction*). The unstandardised regression coefficient (β) represents the estimated change in the pooled treatment effect size (standardised mean difference; SMD) associated with a one-unit increase in the moderator variable. For the Sex × Moderator Interaction, positive coefficients indicate greater treatment effects among male participants at higher levels of the moderator.  *k/N* = the number of effect sizes (outcomes) and the number of independent trials (studies) contributing to the analysis.  *I²* = the total heterogeneity across the effect sizes (in percentage).  *β* = the estimated pooled standardised mean difference (SMD) with the standard error (SE) reported in parentheses.  *SE* = the standard error.  *95% CI* = 95% confidence intervals with the lower bound (LB) and upper bound (UB)  *p* = *p*-value associated with the test of the null hypothesis that the effect is zero.  *Baseline Severity* refers to the pre-treatment mean value of the outcome variable.  *Sample Age* represents the average participant age in each study sample  *Treatment Length* indicates the total duration of the intervention in weeks. | | | | | | | | | | | | | | | | | |
