# Supplementary material for: Effectiveness of Alcohol Use Disorder Pharmacotherapies by Sex: Systematic Review and Meta‐Analysis
Source: Drug Alcohol Rev. 2026 Jun 23;45(5):e70196. doi: 10.1111/dar.70196 (PMC13290497; doi:10.1111/dar.70196)
Supplement: Supplementary file 12 — Table S5: Full text exclusions. [file DAR-45-0-s008.docx]

**Full-Text Articles Excluded After Eligibility Assessment, With Reasons (n=33)**

(PRISMA ITEM 16b)

This table lists all reports that underwent full-text review and were subsequently excluded from the final analysis. Reasons for exclusion are provided in accordance with Item 16b of the PRISMA 2020 statement. Each study is listed once with the primary reason for exclusion as determined during eligibility assessment.

| **Table S5. Full Text Exclusions** | | |
| --- | --- | --- |
|  | *Outcomes Not Reported by Sex (n=8)* | |
|  |  | Clark, D. C., & Fawcett, J. (1989). Does lithium carbonate therapy for alcoholism deter relapse drinking? [Review]. Recent developments in alcoholism : an official publication of the American Medical Society on Alcoholism, the Research Society on Alcoholism, and the National Council on Alcoholism, 7, 315-328. <https://www.scopus.com/inward/record.uri?eid=2-s2.0-0024572714&partnerID=40&md5=4151b816f857f7d7743bc08f33a41589> |
|  |  | Kranzler, H. R., Burleson, J. A., Korner, P., Del Boca, F. K., Bohn, M. J., Brown, J., & Liebowitz, N. (1995). Placebo-controlled trial of fluoxetine as an adjunct to relapse prevention in alcoholics. The American Journal of Psychiatry, 152(3), 391-397. <https://doi.org/https://doi.org/10.1176/ajp.152.3.391> |
|  |  | Martinotti, G., Romanelli, R., Di Nicola, M., Reina, D., Mazza, M., & Janiri, L. (2007). Oxcarbazepine at high dosages for the treatment of alcohol dependence. American Journal on Addictions, 16(3), 247-248. <https://doi.org/10.1080/10550490701375558> |
|  |  | Richter, C., Effenberger, S., Bschor, T., Bonnet, U., Haasen, C., Preuss, U. W., Heinz, A., Forg, A., Volkmar, K., Glauner, T., & Schaefer, M. (2012). Efficacy and Safety of Levetiracetam for the Prevention of Alcohol Relapse in Recently Detoxified Alcohol-Dependent Patients A Randomized Trial. Journal of Clinical Psychopharmacology, 32(4), 558-562. <https://doi.org/10.1097/JCP.0b013e31825e213e> |
|  |  | Guardia, J., Caso, C., Arias, F., Gual, A., Sanahuja, J., RamÃ­rez, M., Mengual, I., Gonzalvo, B., Segura, L., Trujols, J., & Casas, M. (2002). A double-blind, placebo-controlled study of naltrexone in the treatment of alcohol-dependence disorder: Results from a multicenter clinical trial [Article]. Alcoholism: Clinical and Experimental Research, 26(9), 1381-1387. <https://doi.org/10.1097/00000374-200209000-00011> |
|  |  | Oslin, D. W., Lynch, K. G., Pettinati, H. M., Kampman, K. M., Gariti, P., Gelfand, L., Ten Have, T., Wortman, S., Dundon, W., Dackis, C., & et al. (2008). A placebo-controlled randomized clinical trial of naltrexone in the context of different levels of psychosocial intervention [Journal article]. Alcoholism, clinical and experimental research, 32(7), 1299-1308. <https://doi.org/10.1111/j.1530-0277.2008.00698.x> |
|  |  | Johnson, B., Alho, H., Addolorato, G., Lesch, O. M., Chick, J., Liu, L., & Schuyler, V. (2024). Low-dose ondansetron: A candidate prospective precision medicine to treat alcohol use disorder endophenotypes. Eur J Intern Med, 127, 50-62. <https://doi.org/10.1016/j.ejim.2024.06.001> |
|  |  | Tiouririne, N. A. D., Kalelioglu, T., Seneviratne, C., & Wang, X. Q. (2024). Safety and tolerability of topiramate and N-acetyl cysteine combination in individuals with alcohol use disorder: a 12 week, randomized, double-blind, pilot study. Alcohol and alcoholism, 59(2 C7 - agad082). <https://doi.org/10.1093/alcalc/agad082> |
|  | *Parent Paper to Included Record (n=6)* | |
|  |  | Naranjo, C. A., Bremner, K. E., & Lanctot, K. L. (1995). Effects of citalopram and a brief psyche-social intervention on alcohol intake, dependence and problems [Journal article]. Addiction., 90(1), 87-99. <https://www.cochranelibrary.com/central/doi/10.1002/central/CN-00172286/full> |
|  |  | Yoon, G., Kim, S. W., Thuras, P., & Westermeyer, J. (2011). Safety, tolerability, and feasibility of high-dose naltrexone in alcohol dependence: an open-label study. Human Psychopharmacology-Clinical and Experimental, 26(2), 125-132. <https://doi.org/10.1002/hup.1183> |
|  |  | Wiesbeck, G. A., Weijers, H.-G., Lesch, O. M., Glaser, T., Toennes, P.-J., & Boening, J. (2001). Flupenthixol decanoate and relapse prevention in alcoholics: Results from a placebo-conrolled study. Alcohol and Alcoholism, 36(4), 329-334. <https://doi.org/https://doi.org/10.1093/alcalc/36.4.329> |
|  |  | Suh, J. J., Pettinati, H. M., Kampman, K. M., & O'Brien, C. P. (2008). Gender Differences in Predictors of Treatment Attrition with High Dose Naltrexone in Cocaine and Alcohol Dependence. American Journal on Addictions, 17(6), 463-468, Article Pii 906011123. <https://doi.org/10.1080/10550490802409074> |
|  |  | Logge, W., Baillie, A., Haber, P., Towers, E., Riordan, B. C., & Morley, K. (2023). Sex differences in the interrelations between stress, craving and alcohol consumption across individuals and time during baclofen treatment for alcohol dependence. Addictive Behaviors, 136, Article 107462. <https://doi.org/10.1016/j.addbeh.2022.107462> |
|  |  | Pettinati, H. M., Silverman, B. L., Battisti, J. J., Forman, R., Schweizer, E., & Gastfriend, D. R. (2011). Efficacy of Extended-Release Naltrexone in Patients with Relatively Higher Severity of Alcohol Dependence. Alcoholism-Clinical and Experimental Research, 35(10), 1804-1811. <https://doi.org/10.1111/j.1530-0277.2011.01524.x> |
|  | *Incomplete/Varying Pharmacotherapy Exposure (n=5)* | |
|  |  | Collins, G. B., Janesz, J. W., Byerly-Thrope, J., Forsythe, S. B., & Messina, M. J. (1985). The Cleveland Clinic Alcohol Rehabilitation Program: A treatment outcome study. A preliminary report [Article]. Cleveland Clinic Quarterly, 52(2), 245-251. <https://doi.org/10.3949/ccjm.52.2.245> |
|  |  | Aguiar, P., Neto, D., Lambaz, R., Chick, J., & Ferrinho, P. (2012). Prognostic Factors During Outpatient Treatment for Alcohol Dependence: Cohort Study with 6 months of Treatment Follow-up. Alcohol and Alcoholism, 47(6), 702-710. <https://doi.org/10.1093/alcalc/ags097> |
|  |  | Caputo, F., Maremmani, A. G. I., Addolorato, G., Domenicali, M., Zoli, G., D'Amore, A., Maremmani, I., & Bernardi, M. (2016). Sodium oxybate plus nalmefene for the treatment of alcohol use disorder: A case series. Journal of Psychopharmacology, 30(4), 402-409. <https://doi.org/10.1177/0269881116629126> |
|  |  | Croissant, B., Klein, O., Gehrlein, L., Kniest, A., Hermann, D., Diehl, A., & Mann, K. (2006). Quetiapine in relapse prevention in alcoholics suffering from craving and affective symptoms: a case series. European Psychiatry, 21(8), 570-573. <https://doi.org/10.1016/j.eurpsy.2006.04.007> |
|  |  | Dore, G. M., Lo, K., Juckes, L., Bezyan, S., & Latt, N. (2011). Clinical Experience with Baclofen in the Management of Alcohol-Dependent Patients with Psychiatric Comorbidity: A Selected Case Series. Alcohol and Alcoholism, 46(6), 714-720. <https://doi.org/10.1093/alcalc/agr131> |
|  | *No Comparator Group or Pre-Post Measure (n=5)* | |
|  |  | Borup, C., & Unden, M. (1994). Combined fluoxetine and disulfiram treatment of alcoholism with comorbid affective disorders. A naturalistic outcome study, including quality of life measurements [Article]. European Psychiatry, 9(2), 83-89. <https://www.scopus.com/inward/record.uri?eid=2-s2.0-0028347328&partnerID=40&md5=079944278af42edf6fcd8a537960c406> |
|  |  | Pelc, I., Hanak, C., Baert, I., Houtain, C., Lehert, P., Landron, F., & Verbanck, P. (2005). Effect of community nurse follow-up when treating alcohol dependence with acamprosate. Alcohol and Alcoholism, 40(4), 302-307. <https://doi.org/10.1093/alcalc/agh136> |
|  |  | Kolla, B. P., Schneekloth, T. D., Biernacka, J. M., Frye, M. A., Mansukhani, M. P., Hall-Flavin, D. K., Karpyak, V. M., Loukianova, L. L., Lesnick, T. G., & Mrazek, D. (2011). Trazodone and Alcohol Relapse: A Retrospective Study Following Residential Treatment. American Journal on Addictions, 20(6), 525-529. <https://doi.org/10.1111/j.1521-0391.2011.00172.x> |
|  |  | Caputo, F., Trevisan, C., Vignoli, T., Maremmani, A. G. I., Montesano, F., Carboni, G., Lungaro, L., Costanzini, A., Caio, G., Testino, G., Volpato, S., & De Giorgio, R. (2024). Efficacy of sodium oxybate plus disulfiram for the maintenance of alcohol abstinence in treatment-resistant patients with alcohol use disorder: a multicentre retrospective study. Ann Ist Super Sanita, 60(4), 252-257. <https://doi.org/10.4415/ann_24_04_03> |
|  |  | Joseph, J., & Khakha, D. C. (2024). Alcohol Abstinence, Adherence, and Attitudes toward Disulfiram Treatment for Alcohol Dependence among Patients Attending a Tertiary Care Setting in North India. Addict Health, 16(3), 152-158. <https://doi.org/10.34172/ahj.1537> |
|  |  | Borup, C., & Unden, M. (1994). Combined fluoxetine and disulfiram treatment of alcoholism with comorbid affective disorders. A naturalistic outcome study, including quality of life measurements [Article]. European Psychiatry, 9(2), 83-89. <https://www.scopus.com/inward/record.uri?eid=2-s2.0-0028347328&partnerID=40&md5=079944278af42edf6fcd8a537960c406> |
|  | *Outcomes not Applicable (n=3)* | |
|  |  | Addolorato, G., Castelli, E., Stefanini, G. F., Casella, G., Caputo, F., Marsigli, L., Bernardi, M., & Gasbarrini, G. (1996). An open multicentric study evaluating 4-hydroxybutyric acid sodium salt in the medium-term treatment of 179 alcohol dependent subjects [Article]. Alcohol and Alcoholism, 31(4), 341-345. <https://doi.org/10.1093/oxfordjournals.alcalc.a008160> |
|  |  | Herbeck, D. M., Jeter, K. E., Cousins, S. J., Abdelmaksoud, R., & Crevecoeur-MacPhail, D. (2016). Gender differences in treatment and clinical characteristics among patients receiving extended release naltrexone. Journal of Addictive Diseases, 35(4), 305-314. <https://doi.org/10.1080/10550887.2016.1189659> |
|  |  | Kiefer, F., Jahn, H., & Wiedemann, K. (2005). A Neuroendocrinological Hypothesis on Gender Effects of Naltrexone in Relapse Prevention Treatment: Letter to the Editor. Pharmacopsychiatry, 38(4), 184-186. <https://doi.org/https://doi.org/10.1055/s-2005-871244> |
|  | *Too Few Female Participants (n=2)* | |
|  |  | Burnett, G. B., & Reading, H. W. (1970). PHARMACOLOGY OF DISULFIRAM IN TREATMENT OF ALCOHOLISM. British Journal of Addiction, 65(4), 281-288. <https://doi.org/10.1111/j.1360-0443.1970.tb03946.x> |
|  |  | Koppad, G., Suvarna, D., Prabhuswamy, N. H., Venu, A., Vijaykumar, T. R., Gundam, R. R., Ramesh, H. M., Sharathchandra, K. K., Bajaj, D., Kabra, A., Vinodkumar, L., & Mohith, H. N. (2024). Baclofen versus Acamprosate for Maintainence of Alcohol Abstinence in Patients with Cirrhosis of Liver: A Double Blinded Randomized Trial. Surgery, Gastroenterology and Oncology, 28, s24-s30. <https://doi.org/10.21614/sgo-615> |
|  | *Pooled Analysis but More Recent Record Included (n=2)* | |
|  |  | Verheul, R., Lehert, P., Geerlings, P., Koeter, M. W. J., & van den Brink, W. (2005). Predictors of acamprosate efficacy: results from a pooled analysis of seven European trials including 1485 alcohol-dependent patients. Psychopharmacology, 178(2-3), 167-173. <https://doi.org/10.1007/s00213-004-1991-7> |
|  |  | Koeter, M. W., van den Brink, W., & Lehert, P. (2010). Effect of early and late compliance on the effectiveness of acamprosate in the treatment of alcohol dependence [Journal article]. Journal of Substance Abuse Treatment, 39(3), 218-226. <https://doi.org/10.1016/j.jsat.2010.06.002> |
|  | *Non-Clinical Sample (n=1)* | |
|  |  | Varshney, M., Kaur, A., Sarin, S. K., Shasthry, S. M., & Arora, V. (2025). Safety and Effectiveness of Naltrexone in the Management of Alcohol Use Disorder in Patients With Alcohol-associated Cirrhosis: First Clinical Observation From Indian Cohort. Journal of Clinical and Experimental Hepatology, 15(2 C7 - 102447). <https://doi.org/10.1016/j.jceh.2024.102447> |
|  | *Unknown Sample Sex (n=1)* | |
|  |  | Kulkarni, A. (2023). BACLOFEN'S EFFECTIVENESS AND SAFETY IN TREATING ALCOHOL USE DISORDER: a RANDOMIZED, DOUBLE-BLIND, PLACEBO-CONTROLLED TRIAL. International journal of academic medicine and pharmacy, 5(4), 237-240. <https://doi.org/10.47009/jamp.2023.5.4.49> |
